# Fine-tuning autophagy maximises lifespan and is associated with changes in mitochondrial gene expression in *Drosophila*

**DOI:** 10.1371/journal.pgen.1009083

**Published:** 2020-11-30

**Authors:** Ivana Bjedov, Helena M. Cochemé, Andrea Foley, Daniela Wieser, Nathaniel S. Woodling, Jorge Iván Castillo-Quan, Povilas Norvaisas, Celia Lujan, Jennifer C. Regan, Janne M. Toivonen, Michael P. Murphy, Janet Thornton, Kerri J. Kinghorn, Thomas P. Neufeld, Filipe Cabreiro, Linda Partridge

**Affiliations:** 1 Institute of Healthy Ageing, Research Department of Genetics, Evolution and Environment, University College London, London, United Kingdom; 2 UCL Cancer Institute, Paul O'Gorman Building, London United Kingdom; 3 Max Planck Institute for Biology of Ageing, Cologne, Germany; 4 MRC London Institute of Medical Sciences, Imperial College London, Du Cane Road, London, United Kingdom; 5 EMBL European Bioinformatics Institute, Wellcome Trust Genome Campus, Hinxton, Cambridge, United Kingdom; 6 Section on Islet Cell and Regenerative Biology, Research Division, Joslin Diabetes Center, Boston MA, United States of America; 7 Department of Genetics and Harvard Stem Cell Institute, Harvard Medical School, Boston MA, United States of America; 8 Institute of Structural and Molecular Biology, University College London, London, United Kingdom; 9 LAGENBIO, Facultad de Veterinaria-IIS, IA2-CITA, CIBERNED, Universidad de Zaragoza, Zaragoza, Spain; 10 MRC Mitochondrial Biology Unit, the Keith Peters Building, University of Cambridge, Cambridge, United Kingdom; 11 Department of Genetics, Cell Biology and Development, University of Minnesota, 321 Church St. SE, Minneapolis, MN, United States of America; Karolinska Institutet, SWEDEN

## Abstract

Increased cellular degradation by autophagy is a feature of many interventions that delay ageing. We report here that increased autophagy is necessary for reduced insulin-like signalling (IIS) to extend lifespan *in Drosophila* and is sufficient on its own to increase lifespan. We first established that the well-characterised lifespan extension associated with deletion of the insulin receptor substrate *chico* was completely abrogated by downregulation of the essential autophagy gene *Atg5*. We next directly induced autophagy by over-expressing the major autophagy kinase Atg1 and found that a mild increase in autophagy extended lifespan. Interestingly, strong Atg1 up-regulation was detrimental to lifespan. Transcriptomic and metabolomic approaches identified specific signatures mediated by varying levels of autophagy in flies. Transcriptional upregulation of mitochondrial-related genes was the signature most specifically associated with mild Atg1 upregulation and extended lifespan, whereas short-lived flies, possessing strong Atg1 overexpression, showed reduced mitochondrial metabolism and up-regulated immune system pathways. Increased proteasomal activity and reduced triacylglycerol levels were features shared by both moderate and high Atg1 overexpression conditions. These contrasting effects of autophagy on ageing and differential metabolic profiles highlight the importance of fine-tuning autophagy levels to achieve optimal healthspan and disease prevention.

## Author summary

The increasing number of people living with age-related diseases underscores the importance of ageing research to improve healthspan. Two well-studied evolutionary conserved interventions that extend lifespan and improve health are dietary restriction and down-regulation of nutrient sensing pathways, such as glucose sensing by insulin and amino acid sensing by the target-of-rapamycin signalling pathway. One common characteristic of these anti-ageing interventions is an increase in autophagy, a cellular pathway that degrades damaged proteins and organelles to supply essential building blocks and energy. To help provide a more direct link between autophagy and healthy ageing, we fine-tuned overexpression of Atg1 kinase, which is critical for autophagy induction, and measured its effect on longevity in the fruit fly *Drosophila*. Interestingly, we observed that a moderate increase in autophagy is beneficial in extending healthy lifespan, whereas strong autophagy up-regulation is detrimental and leads to progressive lipid loss and decreased lifespan. Moderate and stronger Atg1 overexpression displayed opposing transcriptional profiles of mitochondrial genes, being upregulated in long-lived and down-regulated in short-lived Atg1 over-expressing animals. Overall, we provide a detailed description of the phenotypes associated with varying degrees of autophagy up-regulation *in vivo*, demonstrating that autophagy enhancement delays ageing only when applied in moderation.

## Introduction

Ageing is a complex process [1], yet environmental interventions, such as dietary restriction, and genetic alterations that lower insulin and target-of-rapamycin (TOR) signalling, can improve health and extend lifespan in diverse animals [2, 3]. This evolutionary conservation suggests that a better understanding of the ageing process in model organisms can be translated into health benefits in humans. Several mechanisms have been proposed to account for the increased longevity associated with decreased nutrient-sensing signalling, including improved cellular stress responses, elevated levels of repair processes, and alterations in metabolism, mitochondrial physiology and immune responses. However, the optimal target(s) for improving health and preventing disease are still unclear [1]. Interestingly, most of these candidate processes can be modified by macro-autophagy (hereafter referred to as autophagy). This is a cellular ‘self-eating’ process that degrades cellular proteins and defective organelles such as mitochondria [4–7]. Moreover, up-regulation of autophagy is a shared feature of a number of major anti-ageing interventions and lifespan-extending drugs, such as spermidine and rapamycin [6, 8–10]. The role of autophagy as a crucial player in the ageing process is further suggested by the observation that lifespan extension in *C*. *elegans*, either by dietary restriction or by down-regulation of insulin or TOR signalling, is blocked by inhibiting autophagy [9, 11–13].

Autophagy operates at basal levels in all cells to maintain cellular homeostasis and is further induced by stress, such as starvation stress or a lack of growth factors. During autophagy, the activated Atg1 kinase initiates the formation of a pre-autophagosomal structure, which then requires the ubiquitin-activating E1-like enzyme Atg7 to catalyse the conjugation of Atg12 to Atg5, and of Atg8 to phosphatidylethanolamine (PE). This then leads to the formation of an autophagosome, which fuses with a lysosome to degrade its cargo, consisting of proteins, DNA, lipids, glycogen, organelles such as mitochondria, ribosomes, or even entire bacteria [5, 7]. Thus autophagy, by recycling and reorganising the cellular components, is essential for cellular homeostasis and thereby influences all major cellular processes.

In the context of ageing, it is postulated that a decline in autophagy with age may contribute to the ageing process, with a reduction in the ability to clear cellular damage and defective organelles. Indeed, autophagy may have anti-ageing effects via the elimination of dysfunctional mitochondria, whose malfunction may contribute to the ageing process [14–16]. Additional evidence linking autophagy and ageing comes from the observation that the worm orthologue of the transcription factor EB (TFEB), HLH-30, regulates autophagy in *C*. *elegans*, leading to lifespan extension [17].

Although there are considerable indications that modulating autophagy may have anti-ageing effects, direct evidence showing that genetic up-regulation of autophagy extends lifespan is scarce. So far it has been shown that ubiquitous over-expression of Atg5 in mice [18] and neuronal over-expression of Atg8 [19] and Atg1 [20] in flies extends lifespan. In addition, mutant mice exhibiting up-regulation of the autophagy regulator Becn1, through disruption of its interaction with its negative regulator BCL2, display increased longevity [7].

Here we tested whether increasing autophagy in a tissue-specific manner in *Drosophila* can extend lifespan. We demonstrate that mild over-expression of Atg1 in the fat body, intestine and Malpighian tubules induces autophagy and extends lifespan in *Drosophila*. These long-lived flies are lean and sensitive to starvation. They also display up-regulation of genes in mitochondrial-related GO categories, including oxidative phosphorylation. This suggests that increased mitochondrial metabolism may contribute to their longevity. In contrast, we also demonstrate that strong Atg1 overexpression is detrimental, resulting in a rapid decrease of lipid energy stores and shortened lifespan. These short-lived Atg1 over-expressing flies show increased inflammation, inferred from the up-regulation of several anti-microbial peptides and localization of hemocytes to the intestine. The metabolism of these short-lived flies is severely perturbed in association with the down-regulation of mitochondrial–associated gene expression.

In conclusion, we demonstrate that mild autophagy induction can extend lifespan, and is associated with an alteration in mitochondrial metabolism. However, strong Atg1 up-regulation in the same tissues is detrimental to the organism, leading to depletion of energy reserves and impaired tissue homeostasis. These contrasting effects highlight the importance of fine-tuning the levels of autophagy during ageing to optimise health benefits.

## Results

### Autophagy is essential for the lifespan extension of insulin signalling mutant flies

**Fig 1:**
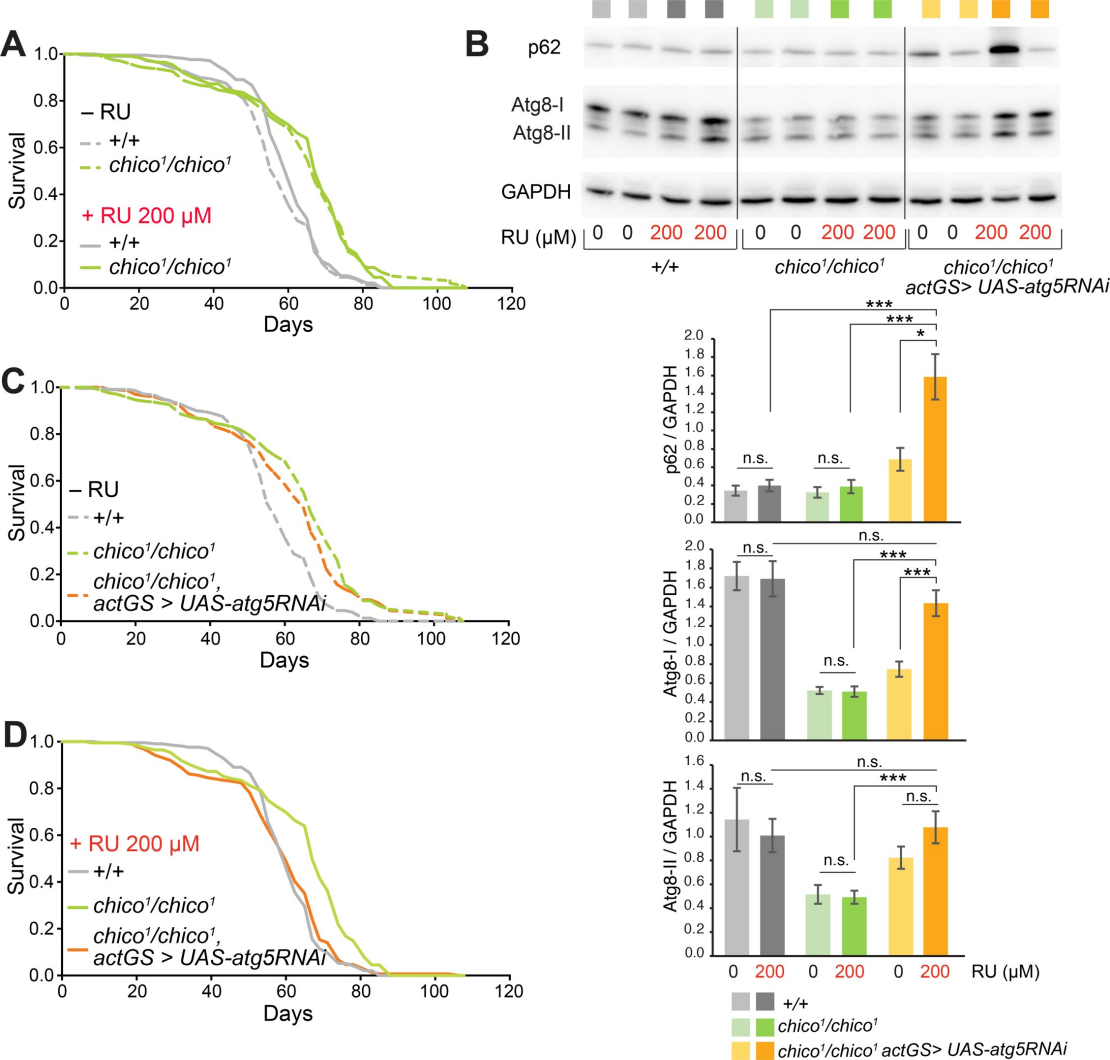
Longevity of *chico*^*1*^ null mutants is abolished upon autophagy down-regulation. (A) c*hico*^*1*^ null mutants are long-lived relative to their *+/+* wild-type controls (p<0.0001, log-rank test comparing genotypes on–RU and +RU). The presence of RU did not affect the lifespan of the c*hico*^*1*^ null and *+/+* controls (p = 0.88 and p = 0.07 respectively, log-rank test comparing ±RU for each genotype). (B) Western blot images and quantification for p62, Atg8a-I and Atg8a-II in control flies, long-lived c*hico*^*1*^ null mutants and c*hico*^*1*^ null mutants with autophagy down-regulated by ubiquitous inducible atg5-RNAi overexpression. A representative Western blot of Atg8a-I (upper band) and Atg8a-II (lower band) levels, with GAPDH as a loading control. Levels of p62 were significantly increased upon down-regulation of autophagy in c*hico*^*1*^/c*hico*^*1*^ *actGS >* UAS-*atg5*RNAi flies relative to the non-induced condition, c*hico*^*1*^ null mutants, and *+/+* controls (p = 0.018, p = 0.0006, p = 0.0006; Student’s *t*-test). Data are means ±SEM of n = 8 replicates. n.s. p>0.05; * p<0.05; *** p<0.001. Each lane is a different biological replicate that was probed for p62, GAPDH and Atg8a. We observed that p62 was variable between different samples while in the same samples Atg8a-I consistently increased upon partial reduction of *Atg5* mRNA. Atg8a-I was significantly higher upon down-regulation of autophagy in c*hico*^*1*^/c*hico*^*1*^ *actGS >* UAS-*atg5*RNAi flies (p = 0.006; Student’s *t*-test; RU versus non-RU and p = 1.8x10^-5^ for comparison with c*hico*^*1*^ null mutant; n = 8). Atg8a-II levels also increased in c*hico*^*1*^/c*hico*^*1*^ *actGS >* UAS-*atg5*RNAi flies compared to c*hico*^*1*^ null mutants (RU condition comparison; p = 0.001; Student’s *t*-test; n = 8). (C) Survival of *+/+* controls, c*hico*^*1*^/c*hico*^*1*^, and c*hico*^*1*^/c*hico*^*1*^ *actGS* > *UAS-atg5*RNAi on standard food (–RU). Both c*hico*^*1*^/c*hico*^*1*^ mutants (±RU) were longer-lived than their *+/+* controls (p<0.0001, log-rank test), but not significantly different from each other (p = 0.15, log-rank test). (D) Ubiquitous down-regulation of autophagy by *Atg5* RNAi abolished the lifespan extension of long-lived *chico*^*1*^ null mutants. Survival curves for wild-type controls (*+/+*), c*hico*^*1*^/c*hico*^*1*^, and c*hico*^*1*^/c*hico*^*1*^ *actGS* > UAS-*atg5*RNAi food on +RU food (200 μM). The c*hico*^*1*^/c*hico*^*1*^ mutant was longer-lived than the *+/+* control (p<0.0001, log-rank test), however the c*hico*^*1*^/c*hico*^*1*^ *actGS* > UAS-*atg5*RNAi mutant was not significantly different from the *+/+* control (p = 0.62, log-rank test) in the presence of RU. n~210 flies per condition for all lifespan experiments.

One of the most robust and well examined anti-ageing interventions is down-regulation of insulin signalling [1]. We examined whether increased autophagy was required for the longevity of insulin receptor substrate (IRS) mutant flies. To do this we used flies homozygous for *chico*^*1*^, a null mutation in the gene encoding the single *Drosophila* IRS homologue, and a well-established long-lived insulin pathway mutant [21, 22]. We first confirmed the longevity of *chico*^*1*^ null flies under our conditions, and also that their lifespan and that of wild-type controls was not affected by treatment with RU (RU486/Mifepristone), the agent used to induce the *Drosophila* GeneSwitch system (Fig 1A) [23]. To test whether enhanced autophagy is required for the longevity of *chico*^*1*^ null flies, we employed RNAi against *Atg5*, which is an essential gene for autophagosome formation [5]. We over-expressed the *UAS-Atg5RNAi* construct ubiquitously in adult flies using the RU-inducible actin-GeneSwitch (*actGS*) driver, in order to circumvent any confounding effects of autophagy inhibition during fly development. This led to transcriptional down-regulation of *Atg5* by 37% in *chico*^*1*^ null flies, as determined by quantitative qRT-PCR (S1A Fig). We measured the autophagy status in *chico*^*1*^ null flies by quantifying levels of the Atg8 and p62 proteins. Atg8 exists in two forms: phosphatidylethanolamine (PE)-modified Atg8a-II, which associates with autophagosomal membranes and positively correlates with the autophagy process, and the unlipidated Atg8a-I, which corresponds to the soluble cytosolic pool. The p62 protein is an autophagy receptor that is degraded by autophagy, hence it negatively correlates with autophagy, and is a commonly used marker for autophagic flux. In *chico*^*1*^ null flies p62 levels remained unchanged compared to controls, which indicates that their autophagy flux is unaltered, despite lower levels of both Atg8a-I and Atg8a-II levels (Fig 1B). This was unexpected, and we hypothesise that in healthy long-lived *chico*^*1*^ null animals there might be a lower requirement for autophagy, or perhaps there are spatiotemporal differences in autophagy that we failed to capture in our experimental approach. Alternatively, the lack of change in p62 protein levels in *chico*^*1*^ mutants can also be due to transcriptional upregulation compensating for increased protein turnover, as has been observed previously during starvation in mammalian cell culture experiments [24]. In support of this, previous transcriptomic studies have observed p62 upregulation in long-lived *chico*^*1*^ flies [25]. Subsequent western blot analyses confirmed efficacy of the *Atg5* RNAi line in reducing autophagy levels, as shown by increased Atg8a-I and p62 levels in both *chico*^*1*^*/chico*^*1*^ *actGS>UAS-atg5RNAi* double mutants (Fig 1B) and flies ubiquitously expressing RNAi against Atg5 (S1B Fig). Lack of down-regulation of Atg8a-II upon *Atg5* downregulation is likely due to its incomplete knockdown (S1A Fig).

Interestingly, the lifespan extension of the *chico*^*1*^ null mutants was completely abolished by down-regulation of autophagy using the *Atg5* RNAi construct (Fig 1C and 1D). Using a further RNAi construct directed against *Atg12*, we observed a similar tendency to reduce *chico*^*1*^ null longevity, although the effects were not significant (S1C–S1F Fig). In wild type flies, reducing autophagy by *actGS>UAS-atg5RNAi* did not alter longevity (S1G Fig). This is in agreement with previous studies in flies showing that only in the presence of bacterial infection does autophagy reduction decrease lifespan [26].

In conclusion, consistent with several studies in worms [9, 11, 27], we show that autophagy is required for the lifespan extension of an insulin signalling mutant in *Drosophila*, highlighting the importance of autophagy as a longevity assurance mechanism. To further investigate the role of autophagy in ageing we next upregulated this process directly.

### Moderate over-expression of *Atg1* in a combination of metabolic tissues produces optimal lifespan extension

To confirm the role of autophagy in ageing, we directly modulated autophagy by over-expressing Atg1, a kinase essential for the initiating steps of autophagy. Atg1 is regulated by the TOR pathway, and its up-regulation is sufficient to initiate autophagy in flies [28, 29]. We tested a variety of GAL4 drivers, both ubiquitous and tissue specific, as well as constitutive and inducible, in order to thoroughly examine the effect of enhanced autophagy on lifespan. We found that over-expression of Atg1 under the control of strong constitutive drivers such as *actinGAL4* (*actGAL4*) and *daughterlessGAL4* (*daGAL4*) was embryonic lethal, presumably due to excessive and inappropriate autophagy during development. Using a weak ubiquitous *heatshockGAL4* (hsGAL4) driver to overexpress *Atg1*, we did not observe any lifespan differences compared to controls. Given that induction of Atg1 can induce apoptotic cell death [28], we next simultaneously overexpressed both Atg1 and the apoptosis inhibitor p35 using hsGAL4. However, this worsened survival, demonstrating that cell death may not be the factor hindering lifespan extension under our conditions (S2A Fig). Next, we restricted the overexpression of Atg1 to adulthood by using the inducible *actGS* driver. However, this resulted in lifespan shortening (S2B Fig), suggesting that induction of autophagy under a strong and ubiquitous promoter was harmful.

To find a tissue that is responsive to autophagy, and in which increased autophagy mediates pro-longevity benefits to the whole organism, we focused our attention on the major metabolic tissues of the fly, since alterations in metabolism and energy storage are characteristic of long-lived mutants [1]. In addition, over-expression of the transcription factor FOXO in the fat body (the fly equivalent of the liver and adipose tissue) and/or the intestine has already been associated with longevity effects in flies and worms [30–33].

**Fig 2:**
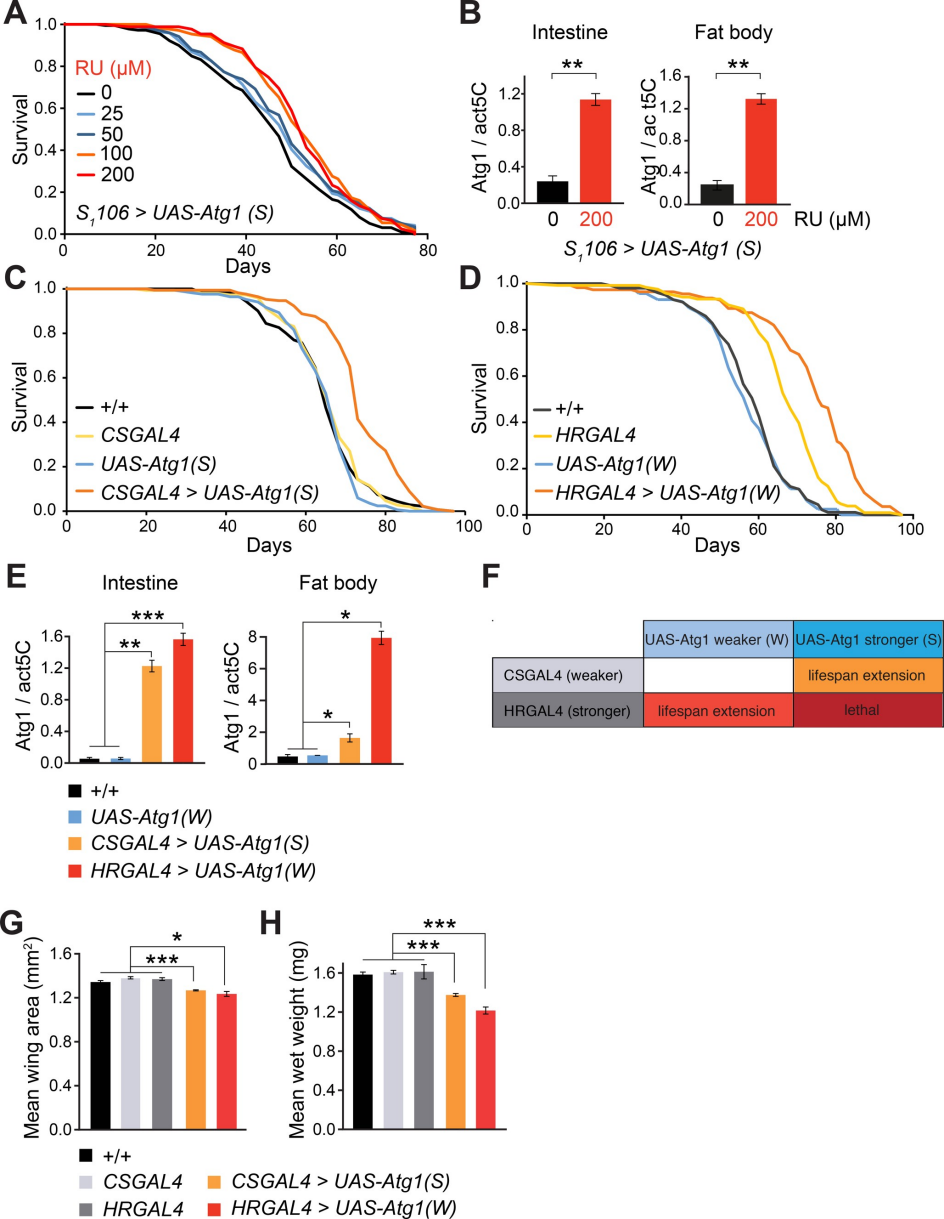
Over-expression of *Atg1* in the gut, fat body and Malpighian tubules extends lifespan. (A) Over-expression of Atg1 in the gut and fat body under control of the inducible *S*_*1*_*106* GeneSwitch driver using a range of RU concentrations. Lifespan was not significantly extended at the lowest RU dose (25 μM; p = 0.076, log-rank test against the 0 μM control), but all higher RU concentrations (50, 100 and 200 μM) significantly increased lifespan (p = 0.00015, p<0.0001, p<0.0001 respectively, log-rank tests against the 0 μM RU control). n~180 flies per condition. (B) Increased transcription of *Atg1* in *S*_*1*_*106 > UAS-Atg1* flies upon induction with 200 μM RU, in the intestine (p = 0.008, Student’s *t*-test) and fat body (p = 0.007, Student’s *t*-test), as determined by qRT-PCR from dissected tissues normalised to *actin5C*. Data are means ±SEM of n = 3 samples (**, p<0.01). (C) Over-expression of *UAS-Atg1(S)* under control of the *CSGAL4* driver significantly extended lifespan (p<0.0001, log rank test against all three control lines). n~120 flies per condition. *CSGAL4* drives expression in the intestine, fat body and Malpighian tubules. (D) Over-expression of *UAS-Atg1(W)* under control of the *HRGAL4* driver significantly extended lifespan (p<0.0001, log rank test against all three control lines). n~120 flies per condition. *HRGAL4* is a stronger driver than *CSGAL4* and they both have the same expression pattern, the intestine, fat body and Malpighian tubules. (E) Expression levels of *Atg1* in the intestine and fat body driven by *HRGAL4* and *CSGAL4*, as determined by qRT-PCR normalised to *actin5C*. Data are means ±SEM of n = 3 samples. Statistical significance was determined by a one-way ANOVA Student’s *t*-test (*, p<0.05; **, p<0.01; ***, p<0.001). (F) Table illustrating different strengths of the drivers and the UAS lines used to overexpress Atg1. (G) Wing surface area was significantly decreased in Atg1 over-expressing flies compared to controls. Data are means ±SEM of n = 10 replicates. Statistical significance was determined by a one-way ANOVA Student’s *t*-test (*, p<0.05; ***, p<0.001). (H) Wet body weight was significantly decreased in Atg1 over-expressing flies compared to controls (p<0.001 and p<0.0001 for the weaker and stronger autophagy enhanced flies, respectively). Data are means ±SEM of n = 6 replicates. Statistical significance was determined by a one-way ANOVA Student’s *t*-test (***, p<0.001).

In order to more finely modulate the level of Atg1 up-regulation, we used two different UAS constructs, *UAS-Atg1(W)* and *UAS-Atg1(S)*, mediating weak and strong over-expression of Atg1, respectively. These UAS constructs were combined with a range of tissue-specific drivers in the fly and screened for lifespan extension. Induction of autophagy using the strong *UAS-Atg1(S)* construct in both the fat body and intestine with the RU-inducible GeneSwitch *S*_*1*_*106* driver led to a dose-dependent lifespan extension (Fig 2A) and increased autophagy flux, as measured by the GFP-p62 cleavage assay (S2C Fig). However, interestingly, we were unable to recapitulate this longevity phenotype using drivers specific to individual tissues, notably *NP1GAL4* (intestine), *FBGAL4* (fat body) and *UOGAL4* (Malpighian tubules, the equivalent of the fly kidney) (S2D–S2H Fig). When we used the *UOGAL4* driver to induce autophagy with either the weaker *UAS-Atg1(W)* or stronger *UAS-Atg1(S)* construct, neither increased fly survival (S2D Fig). Under our experimental conditions, the *NP1GAL4* and *FBGAL4* drivers were lethal in combination with the stronger *UAS-Atg1(S)* and did not affect lifespan when combined with the weaker *UAS-Atg1(W)* (S2E–S2H Fig). Furthermore, over-expression of *UAS-Atg1(S)* only in adulthood using the RU-inducible GeneSwitch gut specific driver *TIGS-2* did not affect lifespan, despite testing a wide range of RU concentrations from 25 μM to 200 μM (S2I and S2J Fig).

These results suggest that autophagy may need to be up-regulated in a combination of tissues to achieve optimal anti-ageing effects, similar to what we observed using the *S*_*1*_*106* driver that is active in both the intestine and the fat body (Fig 2A and 2B). We tested this hypothesis by using the weak *CSGAL4* and strong *HRGAL4* drivers, both of which express in the fat body, intestine and Malpighian tubules (S3A Fig). Accordingly, *UAS-Atg1(S)* driven by weaker *CSGAL4* and *UAS-Atg1(W)* driven by stronger *HRGAL4* both significantly extended lifespan (Fig 2C–2F). We also observed that these long-lived Atg1 over-expressing flies (*CSGAL4* > *UAS-Atg1(S)* and *HRGAL4* > *UAS-Atg1(W)*) had extended egg-to-adult development time, with adults emerging 24 and 65 hours later than wild-type respectively (S3B Fig). Furthermore, the long-lived Atg1 over-expressing flies were slightly smaller than controls, as indicated by their reduced wing area (Fig 2G) and body weight (Fig 2H). Importantly, higher autophagy induction, achieved by combining both the stronger UAS construct and stronger driver lines (*HRGAL4 > UAS-Atg1(S);* Fig 2F), led to embryonic lethality, highlighting that excessive autophagy during development is detrimental, in agreement with previous findings [34].

Next we investigated the importance of the kinase activity of Atg1, since this protein has both kinase-dependent and -independent roles in autophagy (reviewed in [35]). Over-expressing a kinase dead version of Atg1 (*UAS-Atg1*^*KQ*^) driven by *CSGAL4* did not extend lifespan (S3C Fig), suggesting that the kinase activity of Atg1 is necessary for the lifespan extension.

### The pro-longevity effects of moderate autophagy induction are independent of developmental effects

**Fig 3:**
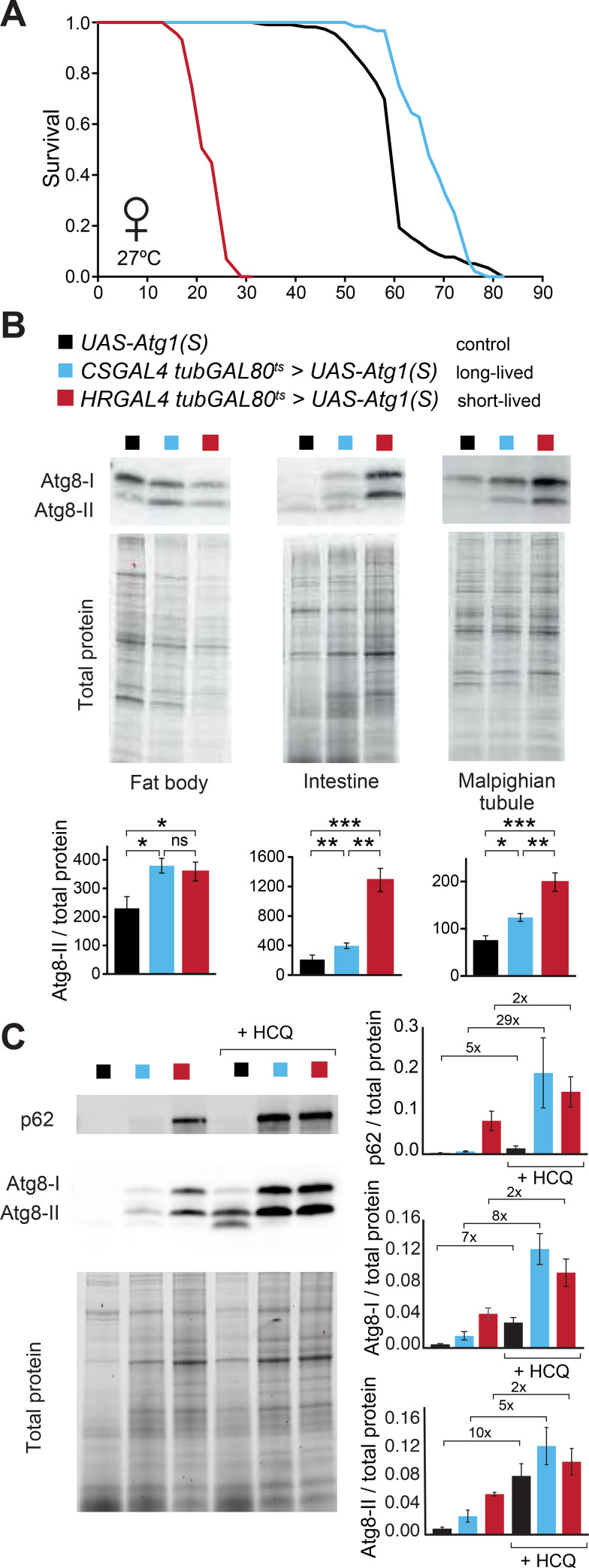
Survival of flies with mild vs. strong autophagy induction. (A) *CSGAL4 tubGAL80*^*ts*^ > *UAS-Atg1(S)* flies were long-lived (p≤0.0001, log rank test), while *HRGAL4 tubGAL80*^*ts*^ > *UAS-Atg1(S)* flies were short-lived (p<0.0001, log rank test) compared to their corresponding driver controls. These lifespan experiments were performed at 27°C to inactivate the *tubGAL80*^*ts*^. n~100 flies per condition. (B) Western blot images of Atg8a-I (upper band) and Atg8a-II (lower band) levels in dissected intestine, fat body and Malpighian tubules, with total protein as a loading control. Over-expression of Atg1 resulted in increased Atg8a-II levels relative to controls in both the long-lived and short-lived autophagy enhanced flies. Data are means ±SEM of n = 4 replicates. Statistical significance was determined by a one-way ANOVA Tukey-Kramer HSD test (*, p<0.05; **, p<0.01; ***, p<0.001). (C) Western blot analysis of p62 and Atg8 using intestinal tissue upon treatment with the autophagy inhibitor hydroxychloroquine (HCQ). Fold increase in band intensity upon HCQ treatment is shown. There was a significant increase of Atg8a-I band intensity upon HCQ treatment for control, *CSGAL4 tubGAL80*^*ts*^>*UAS-Atg1(S)* flies and *HRGAL4 tubGAL80*^*ts*^>*UAS-Atg1(S)* (p = 0.006; p = 0.002; 0.03, respectively, Student’s *t*-test). For Atg8a-II comparisons of non-treated and HCQ-treated condition for controls, long-lived and short-lived flies, p values were 0.006, 0.01 and 0.046; respectively; Student’s *t*-test). Data are means ±SEM of n = 4 replicates.

As over-expression of Atg1 in the fat body, intestine and Malpighian tubules, using the *CSGAL4* and *HRGAL4* drivers, resulted in the most pronounced lifespan extension, we focused on these Atg1 over-expressing flies to explore the mechanisms underlying autophagy-mediated lifespan extension. To avoid any developmental defects, we used the temperature sensitive GAL4 suppressor *tubGAL80*^*ts*^. Flies therefore developed normally at 18°C and were then switched to 27°C as 2-day old adults in order to inactivate GAL80^ts^ and thus enable Atg1 overexpression. By confining Atg1 overexpression to adulthood, we avoided any developmental delay and obtained normal-sized flies, as determined by their wing size (S3D Fig). Moderate autophagy induction in the *CSGAL4 tub-GAL80*^*ts*^ *> UAS-Atg1(S*) flies resulted in lifespan extension (Fig 3A), confirming that the pro-longevity effect was not associated with developmental delay. Interestingly, the otherwise lethal *HRGAL4* > *UAS-Atg1(S)* over-expressing line was viable when combined with flies expressing the *tubGAL80*^*ts*^. However, the stronger autophagy induction obtained in this line did not provide any further benefits, and in fact shortened lifespan significantly, reaching a median survival of only ~25 days at 27°C (Fig 3A). Therefore, for beneficial effects on lifespan, it is necessary to have moderate autophagy induction specifically in a combination of tissues in adulthood. Furthermore, strong overexpression of Atg1 is deleterious not only during development, but also when confined to adulthood.

We were next interested to understand why different degrees of autophagy stimulation lead to such strikingly different outcomes for survival. We used the long-lived *CSGAL4 tubGAL80*^*ts*^ > *UAS-Atg1(S*) flies, with moderate autophagy induction, and the short-lived *HRGAL4 tubGAL80*^*ts*^ > *UAS-Atg1(S)* flies, with strong autophagy induction, for all subsequent experiments. We assessed autophagy induction in the relevant tissues by quantifying Atg8a-II levels in dissected intestine, fat body and Malpighian tubules using Western blot analysis. Indeed, Atg1 overexpression resulted in increased Atg8a-II protein levels in all three tissues (Fig 3B). In the intestine and Malpighian tubules, the short-lived *HRGAL4 tub-GAL80*^*ts*^ > *UAS-Atg1(S)* flies showed higher Atg8a-II protein levels compared to the long-lived *CSGAL4 tubGAL80*^*ts*^ > *UAS-Atg1(S)* flies (Fig 3B). However, in the fat body, there was no difference in Atg8a-II protein levels between the long-lived and short-lived flies, possibly due to rapid depletion of fat body cells upon strong autophagy up-regulation and consequent lower fat body cell content in the short-lived flies. To obtain more information about autophagic flux, we next measured both p62 and Atg8 upon feeding flies the autophagy inhibitor hydroxychloroquine (HCQ) (Fig 3C). Long-lived Atg1 over-expressing flies had increased ratios of p62 between HCQ and untreated conditions, indicative of increased autophagic flux at baseline. Similarly, Atg8a-I and Atg8a-II accumulated once autophagy was blocked by HCQ, confirming that the long-lived Atg1 over-expressing flies have enhanced autophagy. Surprisingly, short-lived flies with higher Atg1 expression showed reduced autophagic flux, as evidenced by a reduced fold increase in p62, Atg8a-I and Atg8a-II proteins once autophagy was inhibited by HCQ.

Atg1 overexpression has previously been linked to negative feedback on mTOR activity in the larval fat body, leading to lower levels of pS6K, a downstream effector of mTORC1 that positively correlates with its activity [28]. We measured pS6K levels in our flies and found that the pS6K to total S6K ratio was not changed upon Atg1 overexpression in either of the Atg1 over-expressing strains (S3E Fig). However, we observed a pronounced increase in both pS6K and total S6K protein. This is in contrast to the initial observations in *Drosophila* larvae, where pS6K was severely reduced upon Atg1 overexpression. This might indicate that different feedback loops operate in larval tissues, that are actively growing, compared to postmitotic adult cells that try to maintain homeostasis upon Atg1 overexpression.

### Immunity-related genes are up-regulated in short-lived flies with strong *Atg1* overexpression

To uncover how autophagy up-regulation can have either beneficial or detrimental effects on lifespan depending on the strength of its induction, we performed genome-wide transcriptional profiling and an unbiased metabolomic analysis on the long- and short-lived Atg1 over-expressing flies. Our system is unique in allowing us to have side by side comparison of two contrasting longevity effects of mild (*CSGAL4 tubGAL80*^*ts*^ > *UAS-Atg1(S)*) and strong (*HRGAL4 tubGAL80*^*ts*^ > *UAS-Atg1(S)*) Atg1 overexpression. For RNA expression profiling, we extracted RNA from the fat body, intestine and Malpighian tubules, as these were the tissues in which we over-expressed Atg1 to induce autophagy. By using a stringent cut-off point for both the long-lived (adjusted p-value < 5 x 10^−5^) and short-lived (adjusted p-value < 1 x 10^−10^) Atg1 over-expressing lines, we observed 90 and 261 differentially expressed genes respectively. Fifty-three genes were significantly differentially expressed in both long-lived and short-lived groups of flies (p-value 1.31 x 10^−59^), indicating a subset of common transcriptional changes underlying both moderate and strong autophagy up-regulation (S4 Fig, full list of genes is available in S1 Data).

To uncover functionally related gene categories that were differentially expressed in the *Atg1* over-expressing flies compared to controls, we performed Catmap analysis based on the ranked list of differentially expressed genes. We used functional annotations for *Drosophila* Gene Ontology (GO) categories and KEGG (Kyoto Encyclopedia of Genes and Genomes) pathways.

**Fig 4:**
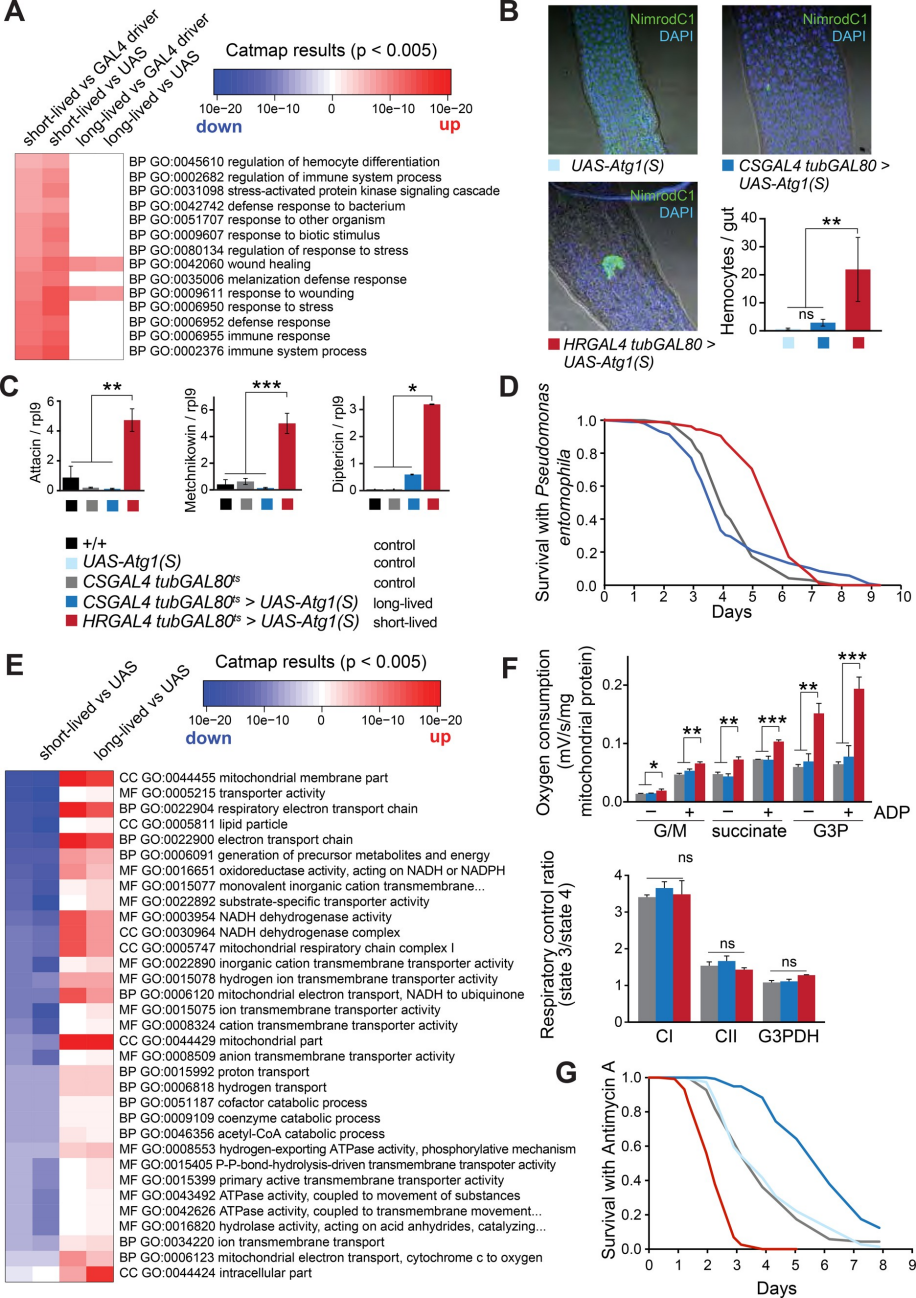
Transcriptomic analysis of the long-lived and short-lived Atg1 over-expressing flies reveals altered immunity and mitochondrial function. (A) Heat map of the immunity- and stress-related GO categories differentially expressed in the long-lived vs. short-lived Atg1 over-expressing flies, showing a pronounced immune and stress response in the short-lived flies only. (B) Hemocyte-specific NimrodC1 staining in the midgut showing increased hemocyte localisation in the short-lived Atg1 over-expressing flies. Quantification of hemocytes in the gut showed increased numbers in the short-lived flies compared to the control and the long-lived flies, according to Student’s *t*-test (**, p<0.01). Data are means ±SEM of n = 10 images. (C) Increased expression of anti-microbial peptides only in the short-lived Atg1 over-expressing flies, as indicated by qRT-PCR for attacin, metchnikowin and diptericin, normalised to *rpl9*. Data are means ±SEM of n = 3 replicates. Statistical significance was calculated by a one-way ANOVA Tukey-Kramer (HSD) test (*, p<0.05; **, p<0.01; *** p<0.001). (D) Stress assay with *Pseudomonas entomophila* showing improved survival of flies with strong enhancement of autophagy (*HRGAL4 tubGAL80*^*ts*^>*UAS-Atg1(S)*) compared to controls and *CSGAL4 tubGAL80*^*ts*^>*UAS-Atg1(S)* flies (p<0.0001, log-rank test, n~100). Survival of control flies and flies with mildly enhanced autophagy was not different (p = 0.88, log-rank test, n~100). (E) Heat map for the mitochondria-related GO categories showing decreased expression of mitochondrial genes in the short-lived flies and increased expression for the same GO categories in the long-lived autophagy flies. (F) Mitochondrial respiration measurements showing increased oxygen consumption with glutamate/malate, succinate and glycerol-3P as a substrate only for the short-lived flies. The long-lived autophagy enhanced flies did not show any difference in respiratory rate compared to the wild-type controls. There was no difference in the respiratory control ratio. Data are means ±range/SD of n = 2–3 measurements and analysed by one-way ANOVA Student’s *t*-test (*, p<0.05; **, p<0.01; ***, p<0.001). (G) Survival upon treatment with the mitochondrial inhibitor antimycin A (0.25 mM). Significant sensitivity or resistance was observed for the short-lived and long-lived Atg1 over-expressing flies, respectively (p<0.001, log rank test, n~150 flies per condition).

First, we explored pathways that were differentially represented in the long-lived compared to the short-lived *Atg1* over-expressing flies, in order to identify autophagy-mediated mechanisms for lifespan regulation. We found that expression of immunity-related GO categories was strongly enriched only in the short-lived Atg1 over-expressing flies (Fig 4A). Namely, categories such as immune response, hemocyte differentiation and defence response to bacterium were strongly up-regulated in the short-lived flies and unchanged in the long-lived flies, with the exception of the wound healing category, which was also increased in the long-lived flies (Fig 4A). To validate these changes, we quantified the number of hemocytes in the intestinal midgut region of *Atg1* over-expressing flies. We hypothesised that strong autophagy in the short-lived flies causes cell death and degradation, which then might stimulate an inflammatory response and attract hemocytes to the site of the lesion [36, 37]. Indeed, staining for the hemocyte-specific marker NimrodC1 [38] revealed a large number of hemocytes in the gut of the short-lived Atg1 over-expressing flies (Fig 4B). Conversely, the long-lived Atg1 over-expressing flies had only a few hemocytes localised throughout the gut, resembling those of wild-type controls (Fig 4B). Furthermore, we measured the expression of several anti-microbial peptides (attacin, diptericin, and metchnikowin) by qRT-PCR (Fig 4C). This revealed that these anti-microbial peptides were strongly up-regulated in flies with excessive autophagy. Interestingly, when these Atg1 over-expressing flies were challenged by feeding *Pseudomonas entomophila* [39, 40], only flies with strong Atg1 up-regulation were resistant (Fig 4D), which is in accordance with the enhanced immunity signature. Moreover, higher attacin and diptericin (Fig 4C) are known to be protective against this orally infectious Gram-negative bacteria [39].

### Mitochondrial-associated genes are up-regulated in long-lived flies with moderate over-expression of *Atg1* and down-regulated in short-lived flies with strong over-expression of *Atg1*

In addition to immunity-related changes, transcriptional analysis uncovered an enrichment of mitochondria-related GO categories between the two groups of *Atg1*-over-expressing flies (Fig 4E). Interestingly, mitochondrial categories responded in opposite directions: moderate Atg1 overexpression led to transcriptional up-regulation of various mitochondrial-related genes, whereas stronger *Atg1* overexpression resulted in down-regulation of the same gene categories. To further our understanding of these mitochondrial changes, we assayed a number of mitochondrial markers. Measurement of mitochondrial DNA (mtDNA) copy number by qRT-PCR demonstrated no changes between the *Atg1* over-expressing flies and controls, suggesting that altered mitochondrial number might not be responsible for the observed transcriptional changes (S5A Fig). This was corroborated by our transcriptional analysis, where no change was detected in peroxisome proliferator-activated receptor-γ-coactivator-1α (PGC1α), the main regulator of mitochondrial biogenesis (Supplementary Data 1). However, we acknowledge we may have missed subtle differences that can only be detected using more sensitive techniques [41]. We then hypothesised that the transcriptional changes may be associated with changes in mitochondrial respiratory chain components. We observed an increase in the protein levels of the subunits NDUFS3 (complex I) and ATP5A (complex V) in the short-lived flies, while their levels in the long-lived flies remained unchanged (S5B Fig). Pyruvate dehydrogenase (PDH), an enzyme that links glycolysis to the TCA cycle by converting pyruvate into acetyl-CoA, showed significantly increased expression in both long-lived and short-lived flies, while cytochrome C was lower in both (S5B Fig). Succinate dehydrogenase (SDHB), which forms mitochondrial complex II, and voltage-selective anion-dependent channel (VDAC), both remained unaltered upon our autophagic alterations (S5B Fig). In summary, while transcriptional changes for mitochondria-related gene processes went in opposite directions between the long- and short-lived flies, there was no clear pattern of differential expression between long-lived and short-lived flies of the mitochondrial specific genes we assessed, with levels of mitochondrial proteins being similarly increased in expression or increased only in the short-lived flies. We cannot however, exclude the possibility that subtle changes in these proteins contribute to the phenotypic changes seen in Atg1 over-expressing flies.

We hypothesised that the differential mitochondrial gene expression might lead to a difference in reactive oxygen species (ROS) production in the short- and long-lived Atg1 over-expressing flies. We therefore assessed mitochondrial hydrogen peroxide (H_2_O_2_) production using the *in vivo* ratiometric mass spectrometry probe, MitoB [42]. Mitochondrial H_2_O_2_ levels were essentially unaffected in the long-lived Atg1 over-expressing flies relative to control (S5C Fig). However, the short-lived Atg1 over-expressing flies had significantly increased levels of mitochondrial H_2_O_2_, suggesting mitochondrial dysfunction.

Next, to gain insight into the respiratory chain activity of these flies, we measured oxygen consumption from isolated mitochondria. The respiratory chain activity of mitochondria from the long-lived *Atg1* over-expressing flies did not differ from control, but was increased in the short-lived flies, when supplied with glutamate/malate, succinate, and particularly glycerol-3-phosphate as the respiratory substrate, although the respiratory control ratios were unaffected (Fig 4F). This was corroborated by metabolomic analysis showing high levels of dihydroxyacetone phosphate, which is produced by glycerol-3-phosphate dehydrogenase, in the short-lived flies (S5D Fig). In addition, higher respiration in the short-lived flies from the complex I-linked substrates glutamate/malate (Fig 4F) are in accordance with increased levels of complex I subunit NDUFS3 measured by western blot (S5B Fig).

To assess how these flies coped with mitochondrial stress at a physiological level, we measured their survival in response to treatment with antimycin A, a well-described inhibitor of mitochondrial respiration. Interestingly, the survival of the long-lived Atg1 over-expressing lines on antimycin A was significantly enhanced, while the short-lived flies were highly sensitive (Fig 4G), demonstrating a clear difference in their mitochondrial robustness.

### *Atg1* overexpression is associated with increased proteasomal activity

**Fig 5:**
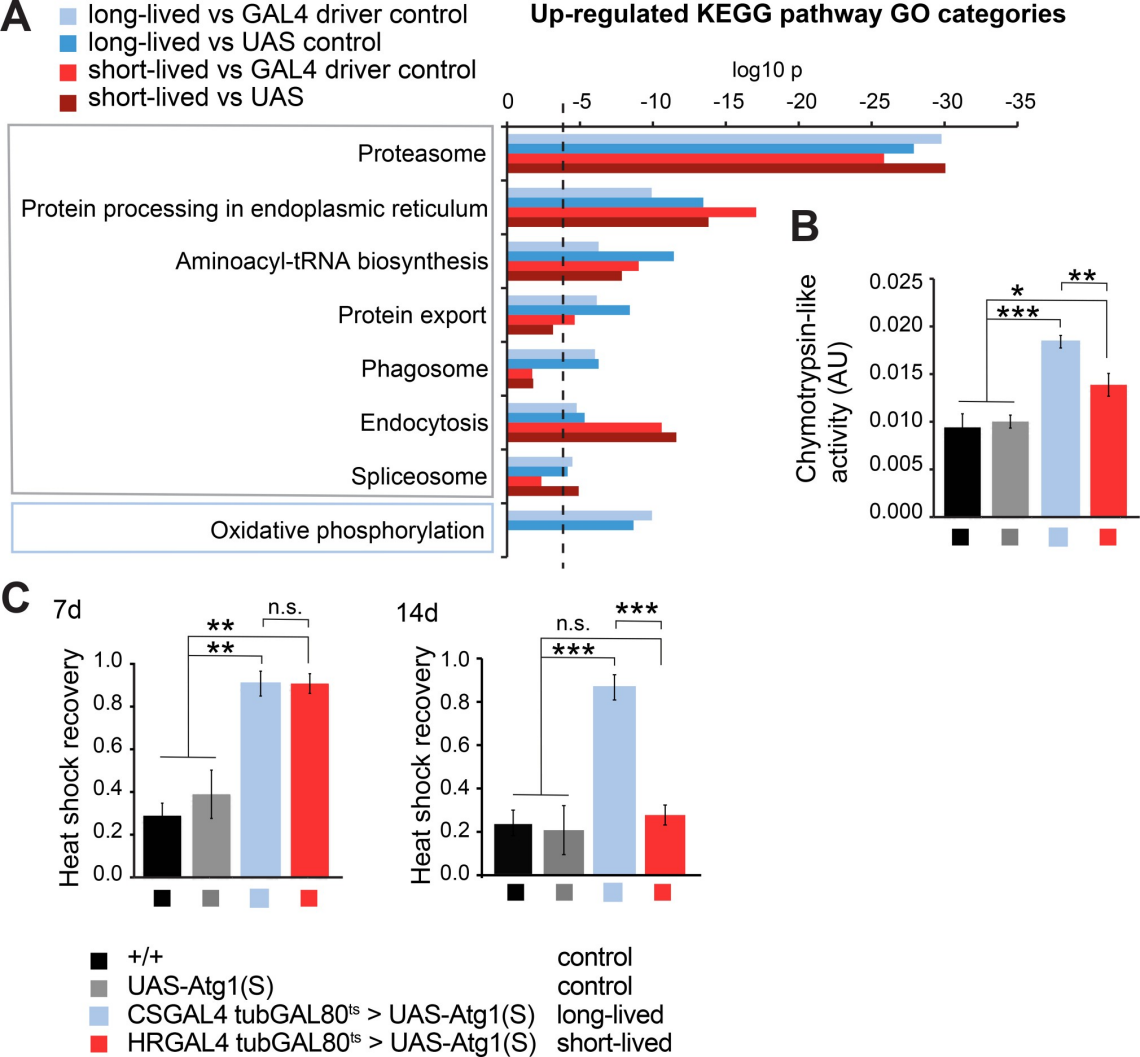
**Transcriptomic analysis of the long-lived and short-lived Atg1 over-expressing lines reveals altered protein homeostasis** (A) List of KEGG pathway GO categories that were up-regulated in the Atg1 over-expressing flies. Similar changes were observed in both mild and strong Atg1 over-expressing, with the exception of oxidative phosphorylation, which was up-regulated only in the long-lived flies having mild Atg1 up-regulation. (B) Proteasome activity was up-regulated in both Atg1 over-expressing flies (chymotrypsin-like, LLVY substrate). Data are means ±SEM of n = 10 replicates. Statistical significance was calculated by one-way ANOVA Student’s *t*-test (*, p<0.05; **, p<0.01; ***, p<0.001). (C) Resistance of 7-day old (left panel) and 14-day old (right panel) flies to heat shock stress, determined as the proportion of flies recovering after 35 min at 39°C. Improved heat shock resistance was observed in both long-lived and short-lived Atg1 over-expressing flies compared to controls for 7-day old flies. Data are means ±SEM of n = 3 samples with 15 flies each. Statistical significance was calculated by one-way ANOVA Student’s *t*-test (n.s., p>0.05; **, p<0.01). In 14-day old flies, the improved heat shock resistance was maintained in the long-lived autophagy flies, but lost in the short-lived (n.s., p>0.05; ***, p<0.001).

Based on KEGG pathway analysis, proteasomal activity was the most pronounced commonly up-regulated category in both *Atg1* over-expressing lines, (Fig 5A). We confirmed this biochemically by assaying chymotrypsin-like proteasomal activity in fly extracts and found that both the long- and short-lived Atg1 over-expressing flies had increased proteasomal activity (Fig 5B). Since worms with increased proteasomal activity are robustly resistant to heat shock stress [43], we exposed the *Atg1* over-expressing lines to heat shock. Indeed, both the long-lived and short-lived *Atg1* over-expressing flies were protected against heat shock stress at one week of age in keeping with the increased proteasomal activity (Fig 5C). However, this heat shock resistance was notably lost from the short-lived flies by two weeks of age, coinciding with their severe deterioration and onset of mortality (Fig 5C).

### *Atg-1* overexpression leads to reduced lipid levels

**Fig 6:**
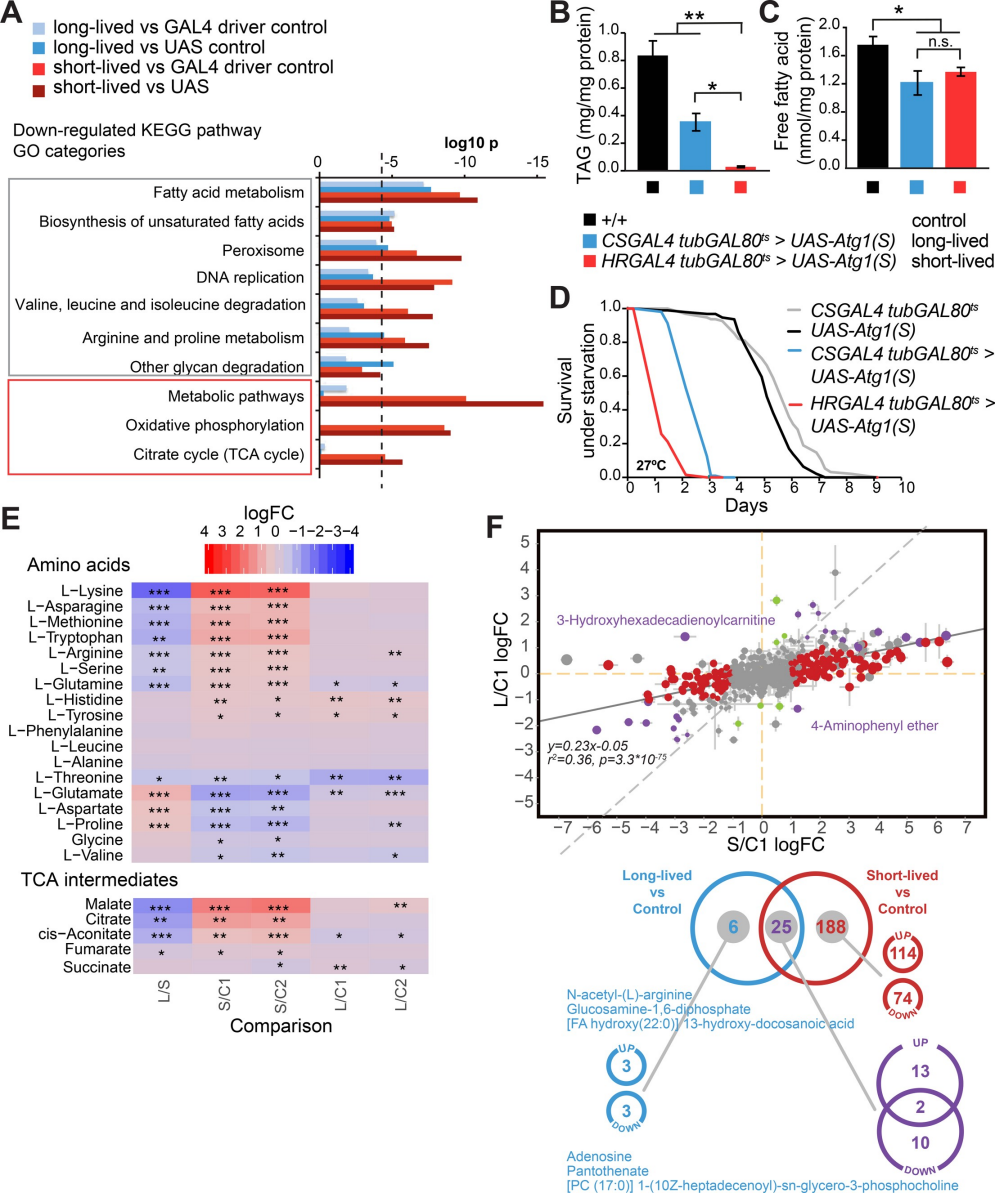
Transcriptomic analysis of the long-lived and short-lived Atg1 over-expressing flies reveals decreased lipid metabolism. (A) The KEGG pathway GO categories that were primarily down-regulated upon autophagy induction were related to lipid metabolism. The trend of transcriptional changes was similar in both the long-lived and short-lived Atg1 over-expressing flies. The categories down-regulated only in the short-lived Atg1 over-expressing flies were metabolic pathways, oxidative phosphorylation, and citrate cycle (TCA cycle). (B) Reduced TAG levels in the autophagy enhanced flies compared to controls. Data are means ±SEM of n = 8 replicates. Statistical significance was calculated by one-way ANOVA Student’s *t*-test (*, p<0.05; **, p<0.01). (C) Reduced free fatty acid levels in both Atg1 over-expressing flies compared to controls. Data are means ±SEM of n = 5 replicates. Statistical significance was calculated by Student’s *t*-test (*, p<0.05). (D) Flies with up-regulated autophagy were significantly more sensitive to starvation stress (p<0.001, log rank test compared to controls). (E) Side-by-side comparison of changes for amino acid and TCA cycle intermediates in Atg1 over-expressing flies. Two controls are used for normalisation: C1 = driver control CSGAL4 tubGAL80^ts^, and C2 = UAS-Atg1(S). Major differences between the long-lived (L) and short-lived (S) flies are represented. Colours show logFC, stars indicate significance: FDR<0.05 = *, FDR<0.01 = **, FDR<0.001 = ***. (F) Comparison of metabolic changes in Atg1 over-expressing flies. Scatterplot visualising the correlation of changes in short-lived and long-lived flies. For illustrative purposes, only the comparison against C1 is shown. Linear regression is provided in the Fig. Two annotated metabolites in the scatterplot change in opposite directions in the Atg1 over-expressing flies. Venn diagram illustrating metabolite changes in Atg1 over-expressing flies that are consistent in comparison to both controls, C1 = CSGAL4 tubGAL80ts, and C2 = UAS-Atg1 (S). Only metabolites with a logFC amplitude >1 and significance of FDR<0.05 are considered. The Venn diagram is further broken down according to the direction of change in a selected subset of data with some of the metabolites highlighted.

One of the most pronounced transcriptional changes in the *Atg1* over-expressing flies was alteration of metabolic pathways (Fig 6A). Autophagy provides cells with amino acids, lipids and other nutrients [7], and is therefore expected to be involved in various aspects of metabolism. In our study, lipid metabolism featured prominently among the down-regulated KEGG pathway categories, including fatty acid metabolism, biosynthesis of unsaturated fatty acids and peroxisome (Fig 6A). Most lipid-related genes were down-regulated in long-lived *Atg1* over-expressing flies, although putative triacylglycerol lipases (CG6283 and CG1882) were both highly expressed in the short-lived Atg1 over-expressing flies (S1 Table). In agreement with the pronounced down-regulation of lipid-related GO categories, we observed that both *Atg1* over-expressing flies had significantly lower levels of triacylglycerides (TAG) with almost total loss of TAG in strong Atg1 over-expressing flies (Fig 6B). Free fatty acids (Fig 6C) were also reduced in both Atg1 over-expressing flies to a similar extent. In keeping with these lipid modifications, both Atg1 over-expressing fly strains displayed pronounced sensitivity to starvation stress (Fig 6D). Oil Red O staining of dissected guts and fat bodies revealed that the *Atg1* over-expressing flies had significantly lower TAG and neutral lipid content compared to controls, with a more pronounced effect in the strong Atg1 over-expressing flies (S5E Fig). This loss of lipid stores was accentuated with age in the short-lived flies (S5E Fig), which progressively lost lipids, ultimately leading to lipid depletion before death. To corroborate other roles of these lipid metabolism-related genes, we performed metabolomic analysis (S6–S8 Figs and Fig 6E and 6F). This demonstrated striking alterations in the metabolic profiles of the Atg1 over-expressing flies, as shown in heat map and principal component analysis (PCA) plots (S6 Fig). Several carnitine metabolites, which are used as lipid oxidation substrates by mitochondria, were consistently found to be strongly up-regulated in long-lived flies compared to short-lived ones (S8 Fig), suggesting increased β-oxidation of lipids as a source of energy, as observed in organisms undergoing dietary restriction.

### Long-lived *Atg-1* over-expressing flies display a unique pro-longevity metabolic profile

Further analysis of the metabolic profiles revealed more pronounced changes in the same metabolites in the short-lived flies than in the long-lived flies (statistically significant correlation between changes in the two autophagy conditions, p = 3.3x10^-75^). Furthermore, we observed a great number of unique changes in the short-lived flies, including amino acids and TCA cycle intermediates, while only a few unique alterations were detected in the long-lived flies (Fig 6E and 6F). There was an overlap of 25 metabolites that changed in both long-lived and short-lived Atg1 over-expressing flies, two of which were in the opposite direction (Fig 6F). Unique to the long-lived flies was an increase in glucosamine-1,6-diphosphate and N-acetyl-(L)-arginine, while levels of adenosine and pantothenate were lower (Fig 6F). Interestingly, food supplementation with different forms of glucosamine, D-glucosamine [44] and N-acetyl glucosamine [45], has been associated with pro-longevity effects. Also, low adenine has been shown to be critical for longevity by dietary restriction and in long-lived AMPK over-expressing flies [46]. Conversely, aged primary cells are associated with greater secretion of adenosine into culture media. [47]. Interestingly, N-acetyl-(L)-arginine and pantothenate, which changed in the opposite direction in our study, are both found to be increased in blood of older human donors and are among 14 metabolites that change the most with age [48]. Therefore, a moderate increase in autophagy is accompanied by several known longevity-promoting metabolite alterations that appear to be absent in short-lived flies with greater Atg1 overexpression.

## Discussion

**Fig 7:**
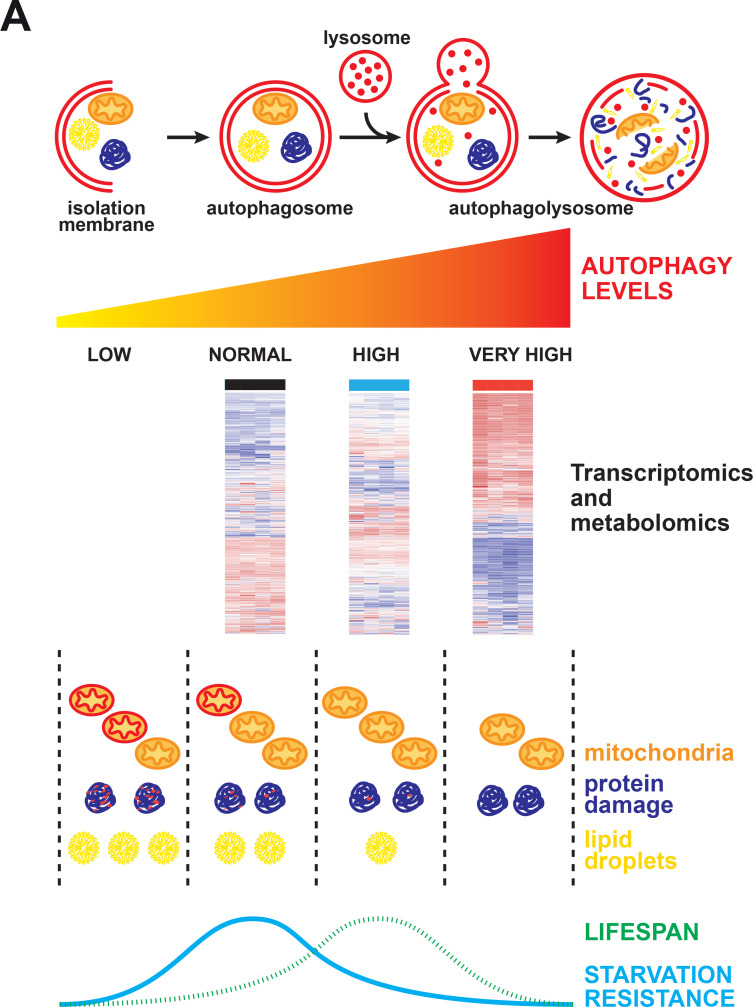
Scheme illustrating the effect of varying Atg1 levels on fly physiology. Organisms with decreased autophagy accumulate damaged macromolecules and organelles, such as mitochondria (damaged mitochondria are represented in red and healthy in orange). Moderate autophagy induction is beneficial for survival, owing to improved damage removal, but leads to compromised survival during starvation, likely as a consequence of the lower lipid content in these flies. Strong Atg1 overexpression does not provide any further longevity benefits, and in fact impairs survival. Moreover, strong Atg1 overexpression is accompanied by induction of an inflammatory response and transcriptional down-regulation of various mitochondrial genes. Overall, the levels of autophagy present in a wild-type organism are optimised for starvation resistance, which is the major stress encountered in nature, rather than for maximised lifespan.

Autophagy in animals evolved primarily to be optimal for survival during starvation, the most common stress in nature. Here, we developed a novel genetic model to enhance autophagy by over-expressing *Atg1* in the major metabolic tissues of the fly and explored in detail its effect on ageing. Our results demonstrate that moderate enhancement of autophagy in a combination of tissues, including the fat body, intestine and Malpighian tubules, is associated with lifespan extension. Conversely, strong or ubiquitous Atg1 expression results in a shortening of lifespan. However, the pro-longevity benefits associated with moderate autophagy induction occur at the expense of survival under starvation, as flies with moderate Atg1 up-regulation display a lean phenotype. These findings suggest that fine-tuning the levels of autophagy in different tissues may be essential for interventions that extend organismal lifespan (Fig 7).

Down-regulation of insulin signalling extends lifespan in all organisms tested, making it one of the best studied and most evolutionarily conserved anti-ageing interventions [3]. Here we found that the lifespan extension of *chico*^*1*^ null flies depends on autophagy, since the longevity of this insulin pathway mutant was abolished in an autophagy-deficient background achieved through RNAi against *atg5*. Long-lived *daf-2* worms similarly require active autophagy for enhanced lifespan [9, 13, 27, 49], and additional studies in *C*. *elegans* have showed tissue-specific roles for autophagy in muscle and intestinal tissues [27]. However, relatively few studies have directly examined autophagy levels in long-lived IIS mutant mice. Reports of increased LC3-II and decreased p62 protein levels in aged mouse hepatic tissue with liver-specific *Igf-1* deletion [50], a manipulation that extends healthy lifespan [51], suggest that increased autophagy could play a role in some long-lived mice with reduced IIS. Taken together with our results, these studies suggest that a careful examination of autophagy in additional long-lived mutant models may be a fruitful direction for future research.

Interestingly, as well as down-regulation of insulin signalling, some other known anti-ageing interventions (including dietary restriction, down-regulation of the TOR signalling pathways, and the anti-ageing drugs rapamycin and spermidine) share up-regulation of autophagy as a common feature [13]. However, these interventions are pleiotropic, and may lead to other anti-ageing effects besides autophagy, such as decreased translation. Moreover, upstream alterations in the IIS and TOR pathways often result in undesirable side-effects, and long-lived mutant model organisms frequently have increased lipid levels and delayed development [1]. Therefore, in order to clarify the mechanism by which these nutrient signalling pathways extend lifespan, and to eliminate any unfavourable side-effects, it is crucial to examine the down-stream effectors of these anti-ageing interventions.

We therefore studied the effect of varying the extent and distribution of *Atg1* overexpression by using diverse tissue-specific drivers. We found that strong ubiquitous up-regulation of autophagy was detrimental to the organismal lifespan. However, when autophagy was increased solely in the major metabolic tissues of the fly—the fat body, intestine and Malpighian tubules—it resulted in viable, long-lived, stress-resilient adults. Expression of the *Atg1* transgene in the fat body and intestine also led to increased longevity, while induction of autophagy in a single tissue was ineffective. This suggests that for these metabolic tissues, autophagy levels are not optimal for longevity and up-regulation is required for overall lifespan extension. Similarly, over-expression of Atg8 and Atg1 in the fly nervous system can extend lifespan [19, 20]. Furthermore, ubiquitous overexpression of Atg5 [18] or down-regulation of Becn1 activity in mice results in increased lifespan [7]. Interestingly in *C*. *elegans*, lifespan extension was observed with post-reproductive autophagy inhibition using RNAi specifically against genes implicated in the early stages of autophagy, suggesting that increased autophagy induction and/or inefficient completion of the process in aged animals can also be harmful [52].

We also explored the effect of enhancing autophagy only in adulthood, thus bypassing developmental effects, in both long- and short-lived *Atg1* over-expressing flies, with moderate and strong autophagy up-regulation respectively. This demonstrated that the pro-longevity effects associated with moderate autophagy induction were independent of development and were not associated with any developmental delay.

In order to further study the underlying mechanisms mediating the lifespan-extending effects of moderate autophagy induction, we used an unbiased transcriptomic and metabolomic analysis approach. This provided a detailed and comprehensive description of the changes in the long-lived and short-lived *Atg1* over-expressing flies. This tissue-specific transcriptional analysis uncovered many similarities between the two over-expressing lines, but also clearly indicated some pronounced differences. For instance, several immunity-related GO categories were unchanged in the long-lived Atg1 over-expressing flies, but were strongly up-regulated in the short-lived flies. This was manifested in the elevated expression of all three anti-microbial peptides tested, and additionally in the accumulation of hemocytes in the intestine. To the best of our knowledge, this is the first demonstration that high levels of autophagy can contribute to hemocyte accumulation in the intestine, and poses interesting questions regarding the potential role of the autophagy process in intestinal regeneration [36, 37, 53].

Another major difference between the long- and short-lived Atg1 over-expressing flies was the strikingly different transcriptional regulation of various mitochondrial-related GO categories: short-lived flies displayed decreased transcription of mitochondrial genes whereas long-lived flies showed markedly increased transcription of the same gene categories. More specifically, oxidative phosphorylation was significantly up-regulated exclusively in the long-lived Atg1 over-expressing flies, while it was down-regulated in the short-lived flies, together with metabolic pathways and TCA cycle GO categories. One significant result was the resistance against the mitochondrial inhibitor antimycin A seen in the long-lived flies. This demonstrates their mitochondrial robustness and differentiates them from the antimycin A sensitive short-lived Atg1 over-expressing flies, thereby confirming our mitochondrial transcriptional signatures. Detrimental effects of high autophagy levels have also been observed in worms, in the context of serum/glucocorticoid regulated kinase-1 (*sgk-1*) mutants, where increased mitochondrial permeability was observed [54]. However, this likely does not fully explain the short lifespan upon strong Atg1 overexpression in our study, as levels of cytochrome C were lower, and VDAC levels unaltered under our conditions, the latter being contrary to the *sgk-1* mutant study [54](S5B Fig).

Although the role of mitochondria in ageing is a well-researched and debated topic, it is still not entirely clear how mitochondria affect lifespan [1, 55, 56]. For instance, mitochondrial ROS production has been proposed as an explanation for ageing, initially as a pro-ageing factor causing damage accumulation [57], and more recently as an anti-ageing mechanism by eliciting beneficial compensatory pathways and extending lifespan through mitohormesis [58, 59]. These contrasting effects of elevated ROS can be reconciled if there is a threshold level, below which ROS are a beneficial mitohormetic, and above which they become detrimental. The high H_2_O_2_ levels seen in the strong Atg1 over-expressing flies could therefore contribute to their short lifespan.

Our mitochondrial analysis and measurements of oxygen consumption revealed higher activity of mitochondrial complex I and complex II, as well as glycerol-3-phosphate dehydrogenase (G3PDH) only in the short-lived flies. However, interpretation of these results is limited given the fact that we used whole fly tissue for respiration analysis, while transcriptional analysis was done on dissected intestine, fat body and Malpighian tubules, the sites of Atg1 transgene overexpression. Most of the mitochondria in the whole fly come from the flight muscle in the thorax, so any subtle respiration effects in mitochondria from the abdominal tissue, would be masked by the flight muscle mitochondria.

Another potential link between mitochondria and ageing is the observation that dietary restriction can exert some of its pro-longevity effects through increased mitochondrial biogenesis. However, some studies have failed to find increased mitochondrial mRNA or protein levels with dietary restriction [60]. Indeed, the most accessible measures of mitochondrial biogenesis, including PGC1α activity and mitochondrial DNA, mRNA, and protein levels, are not a definitive readout of mitochondrial biogenesis per se, which requires more sensitive techniques such as the use of stable isotopic tracers [41]. The increased mitochondrial mRNA expression, and unchanged mitochondrial DNA content, we observe in our long-lived flies therefore suggest that altered mitochondrial function may underlie some beneficial effects in our long-lived flies, but additional studies are required to specify the exact nature of these mitochondrial changes.

Autophagy was initially perceived as a protein-degrading process. However, the degradation of lipids by autophagy, or lipophagy, upon fasting has been described in mouse liver, where autophagy inhibition leads to lipid accumulation [61]. Lipid metabolism has also been associated with ageing, although the underlying mechanisms are unclear, as long-lived mutants can be either lean (*eat-2* worm model for dietary restriction) or have increased adiposity (*daf-2* long-lived worms). This suggests that the quality of the lipids and the sites of fat deposition, rather than the simple overall lipid content, might play a key role in determining ageing [62, 63]. Interestingly, in *C*. *elegans*, mutants deficient in autophagy are characterised by low lipid content, clearly pointing to a complex and tissue-specific role of autophagy in lipid metabolism [64]. Our study, in addition to Ulgherait and co-workers [20], is one of the first to investigate the role of autophagy up-regulation in lipid metabolism. We observed that both *Atg1* over-expressing lines with increased autophagy were lean, with reduced gut lipid content. While the long-lived flies maintained low levels of lipids with age, the short-lived flies progressively lost total lipid content as measured by Oil Red O staining.

The decrease in lipids observed upon increased autophagy could be caused by increased lipolysis, decreased lipid storage and/or decreased lipid biosynthesis. Further insight into the underlying processes was obtained from our transcriptional analysis, which confirmed that autophagy induction triggers lipid remodelling. In particular, we demonstrated that enhanced autophagy can impact on the expression of various lipases and lead to a distinct lipid profile. In turn, these observed changes in lipases may lead to the release of various lipid-derived signalling molecules, which can potentially alter major cellular processes [65, 66].

Interestingly, one of the transcriptionally down-regulated categories upon autophagy induction is the biosynthesis of unsaturated fatty acids. A decrease in unsaturated fatty acids was shown to correlate with lifespan extension in worms, and the explanation suggested was that a high lipid peroxidation index is detrimental for ageing as it can lead to accumulation of cellular damage [67]. Accumulation of damage is one of the hypotheses and hallmarks of ageing [1], indicating that proteasomal degradation of damaged proteins may play an important role in longevity. In keeping with this, we demonstrated increased proteasome activity with Atg1 overexpression, consistent with previous work showing that genetic over-expression of the RPN-6 subunit of the proteasome alone is sufficient to extend lifespan in worms and ameliorate proteotoxic stress resistance [43]. There is also evidence that inhibition of either one of the major degradation pathways, autophagy or proteasomal activity, leads to compensatory up-regulation of the other pathway [68]. Here, we provide novel evidence of the interdependence of these pathways when autophagy is genetically up-regulated, and show how this can lead to broad up-regulation of cellular proteolysis, a situation similar to what is observed upon TOR inhibition [69].

In conclusion, here we describe a full transcriptomic and metabolomic analysis of long-lived and short-lived flies displaying varying degrees of Atg1 overexpression. We demonstrate that moderate genetic up-regulation of autophagy in a combination of metabolic tissues extends lifespan, while excessive Atg1 overexpression is detrimental to longevity. We also show that moderate autophagy induction is associated with a pro-longevity profile across many cellular pathways. In particular, long-lived Atg1 over-expressing flies display increased mitochondrial gene expression and proteasomal activity, in addition to a lean fat body and gut phenotype. Taken together, our data suggest that careful manipulation of the autophagy process is crucial for health benefits, and that any potential applications of autophagy induction, such as autophagy-stimulating drugs, in treating age-related diseases [70], should be tested cautiously and carefully designed.

## Materials and methods

### Fly stocks and husbandry

Mutants and transgenes were back-crossed into the *white Dahomey* (*w*^*Dah*^) wild-type background for at least eight generations. All stocks were maintained, and all experiments were conducted, at 25°C, except *tubGAL80*^*ts*^ experiments, which were set up at 18°C and then switched to 27°C 2 days after the flies eclosed. Flies were kept on a 12 h light:12 h dark cycle at constant humidity using standard sugar/yeast/agar (SYA) medium [71]. For all experiments, flies were reared at standard larval density by transferring 18 μl of egg suspension into SYA bottles [71]. Eclosing adults were collected over a 12-h period and allowed to mate for 48 h before sorting into single sexes.

The following autophagy lines were used: *UAS-Atg5-RNAi*, *UAS-Atg1*^*KQ*^, *UAS-Atg1(S)* and *UAS-Atg1(W)* [26, 28, 72]. The *UAS-Atg1(S)* line corresponds to *UAS-Atg1*^*6B*^, generated by cloning cDNA (AT02023) into pUAST. The *UAS-Atg1(W)* line corresponds to *UAS-Atg1GS10797* from the Kyoto Drosophila Genetic Resource Center and has a UAS regulatory sequence inserted upstream of the endogenous *atg1* gene. *chico*^*1*^*/Cyo* [73] was a kind gift from Ernst Hafen. The driver lines *actGS* [74], *TIGS-2* [75], *FBGAL4* [76], *UOGAL4* [77] were generous gifts from John Tower, Laurent Seroude, Sebastian Grönke and Julian Dow, respectively. *tubGAL80*^*ts*^ was obtained from the Bloomington *Drosophila* Stock Center. *S*_*1*_*106* is a kind gift from the R. Davis laboratory [78] and *NP1GAL4* was obtained from the Kyoto Drosophila Genetic Resource Center. *HRGAL4* and *CSGAL4* are *6g1*Cs-GAL4-1a and *6g1*HR-GAL4-6c, respectively [79]. For the inducible GeneSwitch system, standard SYA food was supplemented with the drug RU486 (Mifepristone, Sigma) typically at a dose of 200 μM unless otherwise indicated.

### Lifespan assays

Flies reared at standard density were maintained at 10 or 15 flies per vial. Flies were transferred to fresh food vials every 2–3 days and scored for deaths. All lifespan experiments were performed using female flies and were repeated at least twice (except the *chico*^*1*^*/chico*^*1*^ *actGS > UAS-Atg5-RNAi* and *chico*^*1*^*/chico*^*1*^ *actGS > UAS-Atg12-RNAi* lifespan). At least 100 flies were used for each genotype.

### Wing and weight measurements

Wing surface area from 7-day old flies (n>10 per genotype) was measured as previously described [73]. Whole body wet weight (n = 10 per genotype) was measured using a precision balance.

### Stress assays

Flies for stress assays were reared and maintained as for lifespan experiments. For starvation stress, 7-day old flies were transferred to vials containing 1% agar and deaths were scored several times per day. For heat shock stress, 7- or 14-day old flies were incubated at 39°C for 35 min, then scored for recovery. For the antimycin A survival assay, we used fly food containing 0.25 mM antimycin A (Sigma, A8674), which was dissolved in ethanol as a 100 mM stock solution. For oral infection of flies with *Pseudomonas entomophila*, vials containing SYA food without nipagin nor propionic acid was covered with 50 μl of overnight bacterial culture grown in LB media. After approximately 2h, when the liquid was absorbed, flies were transferred to *P*. *entomophila* containing vials and scored for deaths periodically [39].

### Proteasome assay

Dissected fat body, intestine and Malpighian tubules from 2–4 fresh flies were homogenised in 25 mM Tris-HCl (pH 7.5) buffer on ice and centrifuged. Protein concentration in the supernatant was measured using Bradford reagent (Bio-Rad). 20 μg of sample was loaded onto a black 96-well plate with 25 μM of the proteasome substrate N-Succinyl-Leu-Leu-Val-Tyr-7-amido-4-methylcoumarin (LLVY-AMC; Sigma, S6510) in a 200 μl final volume. The excitation/emission wavelengths were 350/440 nm, and enzyme kinetics were recorded at 25°C in a temperature-controlled fluorimetric microplate reader (Tecan Infinite M200). Proteasome activity was quantified using 7-amino-4-methylcoumarin (AMC; Sigma, A9891) as a standard, and determined from the slope of AMC accumulation over time.

### Lipid measurements

To quantify triacylglyceride (TAG) content, 10 batches of two flies were homogenised in 0.05% Tween and assayed using Triglyceride Infinity Reagent (Thermo Scientific, TR22421) as previously described [76]. Free fatty acids were assayed with the Free Fatty Acid Quantification Kit (Abcam, 65341). Measurements were normalised to total protein content as determined using the BCA Protein Assay Kit (Pierce). For Oil Red O staining, dissected guts and fat body tissue were fixed on a microscope slide in 4% formaldehyde (Polysciences, 11814), rinsed in PBS and stained for 20–30 min with a filtered freshly prepared solution of 0.06% Oil Red O in 60% isopropanol. The tissue was mounted in 100% glycerol and imaged.

### Western blots

Whole flies or dissected fly tissue were homogenised in 2x Laemmli loading sample buffer (100 mM Tris pH 6.8, 20% glycerol, 4% SDS; Bio-Rad) containing 50 mM DTT. Extracts were cleared by centrifugation and approximately 20–40 μg of protein extract was loaded per lane on a polyacrylamide gel. Proteins were separated and transferred to a nitrocellulose membrane. The following antibodies were used at the indicated dilutions: β-actin (Abcam, ab8227; 1:4000), Atg8 (a generous gift from Katja Köhler; 1:2000; [80], PDH (Abcam, Ab110334), NDUFS3 (Abcam, ab14711), GAPDH (GeneTex, GTX100118), cytochrome C (BioLegend, 612503, clone 7H8.2C12), SDHB (Abcam, ab14714), ATP5A (Abcam, ab14748), VDAC (Abcam, ab14734). The HRP-conjugated anti-rabbit secondary (Abcam, ab6721; 1:12000) was used. Blots were developed using the ECL detection system (GE, Amersham), and analysed using FIJI software (US National Institutes of Health). To quantify total protein, we used TGX stain-free gels from Bio-Rad (567–8123 or 567–8124) according to the manufacturer’s instructions. For hydroxychloroquine (HCQ) treatment, HCQ was dissolved in water to give a 10M stock. The stock was then added to standard SYA fly food to give a final concentration of 30mM. Flies were pretreated 24h with HCQ before western blot analysis.

### X-Gal staining

X-Gal staining of tissues was carried out as described [81]. Briefly, the dissected tissue was fixed in paraformaldehyde, washed in PBS and in a pre-warmed Fe/NaP buffer before incubation in a standard X-Gal staining solution. After PBS washes the tissue was mounted on a slide with gel/mount solution and imaged.

### Hemocyte staining

For imaging of the gut and hemocytes, flies (n = 10 per genotype) were dissected in cold PBS and fixed for 20–30 min in 4% methanol-free formaldehyde (Polyscience, 11814). After PBST (PBS + 0.1% Triton-X 100) washes, tissues were blocked in 5% donkey serum for 1 h, incubated overnight at 4°C with NimrodC1 primary antibody (a generous gift from István Andó; 1:50), then with secondary antibody for 2 h at room temperature (Life Technologies, A21202; 1:200). Tissues were then mounted in mounting medium (Vectashield Laboratories, H-1200) containing DAPI (1.5 μg/ml) and imaged using a Zeiss LSM 700 confocal microscope.

### Larval development

For larval development experiments, eggs were collected over a period of 3 h. Embryos were allowed to hatch and first instar larvae were hand-picked and transferred to give 25 per vial on standard food. When adult flies started to hatch the number of eclosed Atg1 over-expressing flies and control flies was counted in regular intervals.

### Mitochondrial copy number

Total DNA was extracted from fly abdomens without ovaries (n = 5–10 flies per sample) using the Qiagen Blood and Tissue Kit. Relative mitoDNA was measured by qPCR using primers for mitoDNA: GCTCCTGATATAGCATTCCCACGA and CATGAGCAATTCCAGCGGATAAA; for nucDNA: CGAGGGATACCTGTGAGCAGCTT and GTCACTTCTTGTGCTGCCATCGT. Fast SYBR Green Master Mix (Applied Biosystems) was used according to the manufacturer’s recommendations.

### Mitochondrial respiration

Whole fly mitochondria were isolated as previously described [82]. Oxygen consumption was measured using a 1 ml Clark-type oxygen electrode at 25°C. Mitochondria (0.2 mg protein/ml) were energised with either glutamate/malate (5 mM), succinate (5 mM) or glycerol-3-phosphate (5 mM) as the respiratory substrate. The state 3 rate was determined following addition of ADP (1 mM). The respiratory control ratio (RCR) was calculated from state 3/state 4.

### Mitochondrial H_2_O_2_ levels

*In vivo* mitochondrial H_2_O_2_ levels were measured using the ratiometric mass spectrometry probe MitoB as described previously [83], with injected flies (n = 5, in cohorts of 10) incubated for 4 h at 27°C prior to analysis.

### Statistical analysis

Statistical analysis was performed using JMP software (version 4.0.5; SAS Institute). Log rank tests were performed on lifespan and stress survival curves. Other data were tested for normality using the Shapiro-Wilk W test on studentised residuals and where appropriate log-transformed. One-way analysis of variance (ANOVA) and planned comparisons of means were made using Tukey-Kramer HSD and Student’s *t*-test test.

### Microarray analysis

For Dros2 Affymetrix microarray analysis, the fat body, intestine and Malpighian tubules were dissected from n = 15 flies per sample, with 5 replicates per genotype. To facilitate statistical analysis, each of the autophagy conditions was compared to two controls—the corresponding driver and the *UAS-Atg1(S)* line. Raw data (cel files) were processed to correct for probe-sequence biases, and R’s implementation of the Affymetrix’s MicroArray Suite 5.0 software was used to determine present target transcripts [84]. Data were normalised using LOESS normalisation and a linear model was fitted to identify a set of differentially expressed genes using the R LIMMA package [85]. All individual probes were mapped against all known and predicted transcripts of the *Drosophila melanogaster* genome release version 5.4. FlyBase gene IDs were mapped to Gene Ontology (GO) IDs (version 1.107). For functional analysis using all expressed genes, we used the Wilcoxon rank sum test implemented in Catmap [86]. Ranks of genes were based on the Bayes *t*-statistic for differential expression and, for a given functional category, the significance of the rank sum for all genes in the category was calculated analytically based on a random gene-rank distribution. Array data is deposited in ArrayExpress under the accession number E-MTAB-9391.

### Metabolomics analysis

For metabolomics analysis, 10 whole flies were homogenised with a micropestle in 500 μl of 80% methanol, then sonicated for 1 min and centrifuged for 10 min at 15000 *g*, 4°C. The supernatant was collected and stored at –80°C. Samples were then analysed by hydrophilic interaction liquid chromatography-mass spectrometry (UltiMate 3000 RSLC; Thermo Fisher) with a 150 x 4.6mm ZIC-HILIC column running at 300 μl/min and Orbitrap Exactive (Thermo Fisher). Raw mass spectrometry data were processed using a standard pipeline, consisting of XCMS for peak picking [87], MzMatch for filtering and grouping [88], and IDEOM [89]. Core metabolite identifications were validated against a panel of unambiguous standards by mass and retention time. Additional putative identifications were assigned by mass and predicted retention time [90–92]. Means and SEM were generated for all groups of picked peaks and uploaded to Ingenuity pathway analysis software. Statistical analysis was performed using R. For each metabolite, differences between experimental groups were determined using linear modelling on log2 transformed metabolite peak intensities. Principal component analysis (PCA) was performed using unsupervised multivariate analysis. Significance of differences was evaluated within the linear models using pooled standard errors with the subsequent Benjamini-Hochberg FDR correction for multiple testing. Differences were considered statistically significant when Benjamini-Hochberg FDR < 0.05 as indicated in the Figure, Figure labels or experimental methods. Asterisks denote corresponding statistical significance: *FDR < 0.05; **FDR < 0.01; ***FDR < 0.001, unless stated otherwise. Data are presented as the mean ±SD from at least 3 independent biological replicates, unless stated otherwise in the Figures, Figure labels or experimental methods. Enrichment of KEGG pathways for metabolites was evaluated using log transformed metabolite peak intensities and the “Pathway analysis” tool on MetaboAnalyst 3.0 [93, 94].

### RNA extraction and quantitative RT-qPCR

Total RNA was extracted from whole flies or dissected tissues using TRIzol (GIBCO) according to the manufacturer's instructions. mRNA was reverse transcribed using oligo(dT) primers and the Superscript II system (Invitrogen). RT-qPCR was performed using the Fast SYBR Green Master Mix (Applied Biosystems, 4385612) according to the manufacturer's instructions. Primers were optimised following the procedure from Advanced Biosystems, and relative quantities determined by normalising against *actin5C* (relative standard curve method). Primers used for RT-qPCR were: atg1-F ACCAGAGGCAGAACGCATAC and atg1-R GCAGCCAATTAGCGTAAAGC; atg5-F GACATCCAACCGCTCTGCGCA and atg5-R CAGACGATGACTTCACGTACACC; actin5C-F GAGCGCGGTTACTCTTTCAC and actin5C-R GCCATCTCCTGCTCAAAGTC; attacin-F CCAAGGGCATTGGCAATC and attacin-R TTTCCGGCGGCGAAA; metchnikowin-F GCAACTTAATCTTGGAGCGATT and metchnikowin-R GAAAATGGGTCCCTGGTGA; drosomycin-F CTGCCTGTCCGGAAGATACAA and drosomycin-R TCCCTCCTCCTTGCACACA; rpl9-F CATGATCAAGGGAGTCACGT and rpl9-R ATGTACTTCTCACCCAAGAAG. Primers for attacin, metchnikovin and drosomycin are from reference [95].

## Supporting information

S1 Fig Characterising the effect of autophagy down-regulation on lifespan. (A) Confirmation of decreased *Atg5* transcription upon overexpression of UAS-Atg5 RNAi. Quantification of *Atg5* mRNA levels by qRT-PCR in c*hico*^*1*^/c*hico*^*1*^ *actGS >* UAS-*atg5*RNAi flies ±RU (200 μM). Data are normalised to *actin5C*, and are means ±SEM of n = 3 samples (p = 0.01, Student’s *t*-test, ** p<0.01). (B) Western blot analysis showing an increase in Atg8a-I upon inducing down-regulation of A*tg5* transcription using RU in *actGS >* UAS-*atg5*RNAi flies (p = 0.0002; Student’s *t*-test). Data are means ±SEM of n = 4 samples. (C) Confirmation of decreased *Atg12* transcription upon overexpression of UAS-Atg12 RNAi. Quantification of *Atg12* mRNA levels by qRT-PCR in c*hico*^*1*^/c*hico*^*1*^ *actGS >* UAS-*atg12*RNAi flies ±RU (200 μM). Data are normalised to *actin5C*, and are means ±SEM of n = 3 samples (p = 0.019, Student’s *t*-test, * p<0.05). (D) Survival of *+/+* controls, c*hico*^*1*^/c*hico*^*1*^, and c*hico*^*1*^/c*hico*^*1*^ *actGS* > *UAS-atg12*RNAi on standard food (–RU). Both c*hico*^*1*^/c*hico*^*1*^ mutants were longer-lived than the *+/+* controls (p<0.0001; log-rank test), but not significantly different from each other (p = 0.65, log-rank test). Survival of *+/+* controls and c*hico*^*1*^/c*hico*^*1*^ are same as in Fig 1. (E) Survival of *+/+* controls, c*hico*^*1*^/c*hico*^*1*^, and c*hico*^*1*^/c*hico*^*1*^ *actGS* > *UAS-atg12*RNAi on RU food. Both c*hico*^*1*^/c*hico*^*1*^ mutants were longer-lived than the *+/+* controls (p<0.0001 and p = 0.001; log-rank test), but not significantly different from each other (p = 0.21, log-rank test). (F) Down-regulation of autophagy by *Atg12* RNAi showed a tendency to shorten the lifespan extension of long-lived *chico*^*1*^ null mutants (p = 0.091, log-rank test). Survival curves for c*hico*^*1*^/c*hico*^*1*^ *actGS* > UAS-*atg12*RNAi on control and +RU food (200 μM). n~210 flies per condition for all lifespan experiments. (G) Down-regulation of *Atg5* transcription upon RNAi in the adult flies using inducible *actGS* driver did not affect longevity (p = 0.23, Student’s *t*-test, n~210 flies per condition).
(PDF)

S2 Fig Survival analysis of Atg1 overexpression under the control of different drivers. (A) Constitutive over-expression of *UAS-Atg1(W)* under weak ubiquitous heat shock *hsGAL4* did not alter lifespan compared to driver alone, and lifespan was worsened when overexpression of autophagy was combined with the apoptosis inhibitor p35 (p<0.001, log-rank test comparison of *hsGAL4>UAS-Atg1(W)UAS-p35* with hsGAL4). Inhibition of apoptosis shortened lifespan (p<0.001, log-rank test comparison of *hsGAL4>UAS-p35* with hsGAL4; n~100 flies per condition). (B) Ubiquitous over-expression of *UAS-Atg1(W)* under *actGS*, an inducible ubiquitous GeneSwitch driver, led to lifespan shortening in the presence of 100 μM RU (p<0.001, log-rank test; n~180 flies per condition). (C) GFP-p62 cleavage assay for measurements of autophagy flux demonstrated an apparent decrease in GFP-p62 cleavage in the autophagy flies in the absence of autophagy inhibitor chloroquine but, contrarily, a significant increase in GFP-p62 cleavage under non-saturating levels of chloroquine, suggesting increased flux upon Atg1 overexpression. (p<0.005, Student’s *t*-test). (D) Over-expression of *Atg1* with the Malpighian tubule driver *UOGAL4* did not extend lifespan (p = 0.91 and p = 0.38 for the log-rank test comparison of the *UOGAL4* control with *UOGAL4* > *UAS-Atg1(W)* and *UOGAL4* > *UAS-Atg1(S)* respectively; n~180 flies per condition). (E) Over-expression of Atg1 using the gut driver *NP1GAL4* did not extend lifespan. (p = 0.49, log-rank test comparison of *NP1GAL4> UAS-Atg1(W)* with the wild-type control, n~160 flies per condition). No significant differences were observed between any of the represented lifespans (p>0.05, log-rank test). *NP1GAL4> UAS-Atg1(S)* flies were developmentally lethal. (F) qRT-PCR analysis of *Atg1* expression in the intestine and fat body of flies from (E) showing a statistically significant increase of *Atg1* expression in the intestine of the *NP1GAL4 > UAS-Atg1(W)* flies (p = 0.047, Student’s *t*-test; *, p<0.05) but not in the fat body (p = 0.2638, Student’s *t*-test). (G) Fat body over-expression of *UAS-Atg1(W)* under control of the *FBGAL4* driver did not extend lifespan (p = 0.30, log-rank test comparison of *FBGAL4 > UAS-Atg1(W)* with *FBGAL4*, n~150 flies per condition). No significant differences were observed between any of the represented lifespans (p>0.05, log-rank test). *FBGAL4> UAS-Atg1(S)* flies were developmentally lethal. (H) qRT-PCR analysis of *Atg1* expression in the intestine and fat body of flies from (G) showing a statistically significant increase of *Atg1* expression in the fat body (p = 0.02, Student’s *t*-test; *, p<0.05) but not in the intestine (p = 0.42, Student’s *t*-test) of the *FBGAL4 > UAS-Atg1(W)* flies. (I) No lifespan extension in *TIGS-2* > *UAS-Atg1(S)* flies in the presence of different RU concentrations (p>0.05 for all comparisons with the 0 μM RU control, log-rank test, n~180 flies per condition). *TIGS-2* is an inducible gut-specific GeneSwitch driver. (J) qRT-PCR analysis of *Atg1* expression in the intestine and fat body of flies from (G) showing a statistically significant increase of *Atg1* expression in the intestine of the *TIGS-2 > UAS-Atg1(S)* flies on the 200 μM RU food relative to the 0 μM control (p = 0.0003, Student’s *t*-test; ***, p<0.001), but not in the fat body (p = 0.42, Student’s *t*-test).
(PDF)

S3 Fig Characterisation of the drivers and Atg1 over-expressing flies. (A) Representative X-Gal staining for the *CSGAL4*, *HRGAL4* and *NP1GAL4* drivers. (B) Development time of the autophagy enhanced flies. Flies with increased autophagy by over-expression of *Atg1* driven by *CSGAL4* or *HRGAL4* displayed delayed egg to adult development. n~100 flies per condition. (C) Over-expression of a kinase dead mutant of Atg1, *UAS-Atg1KQ*, driven by *CSGAL4* did not extend lifespan (p = 0.9936, log-rank test comparison of *CSGAL4 > UAS-Atg1KQ* with the *CSGAL4* driver control). The long-lived autophagy enhanced flies *CSGAL4 > UAS-Atg1(S)* was significantly longer lived than the *CSGAL4* control (p<0.0001, log-rank test). n~120 flies per condition. (D) Body size as inferred from wing area measurement in the Atg1 over-expressing flies. Data are means ±SEM of n = 10 females per genotype. There are no statistically significant differences in wing size among the represented genotypes, as calculated by a one-way ANOVA Tukey-Kramer (HSD). (E) Western blot analysis showing no significant change in pS6K/tS6K in Atg1 over-expressing flies compared to controls (*UAS-Atg1(S)* compared to *CSGAL4 tubGAL80*^*ts*^ > *UAS-Atg1(S)* p = 0.76, Student’s t-test; and *UAS-Atg1(S)* compared to *HRGAL4 tubGAL80*^*ts*^ > *UAS-Atg1(S)* p = 0.35, Student’s t-test).
(PDF)

S4 Fig Transcriptional profiling of the Atg1 over-expressing flies. There was significant over-representation of the differentially expressed genes between the two autophagy enhanced lines. The cut-off point of the adjusted p-value was < 5 x 10^−5^ for the long-lived Atg1 over-expressing flies and <1 x 10^−10^ for the short-lived Atg1-over-expressing flies.
(PDF)

S5 Fig Mitochondrial characterisation and Oil Red O staining for lipids of the autophagy enhanced flies. (A) Relative mitochondrial copy number measured by qRT-PCR showed undetectable differences in Atg1 over-expressing flies. (B) Western blot analysis for different mitochondrial proteins showed increased PDH in the long-lived (p = 0.005, Student’s *t*-test) and the short-lived flies (p = 0.037, Student’s *t*-test). Both SDHB levels (p = 0.44 and 0.55 for long-lived and short-lived flies, Student’s *t*-test) and VDAC levels (p = 0.50 and 0.11 for long-lived and short-lived flies, respectively, Student’s *t*-test, n = 9) were unaltered. NDUFS3 and ATP5A were increased in short-lived (p = 0.042 and 0.005, Student’s *t*-test) but not in long-lived flies (p = 0.11 and p = 0.27, Student’s *t*-test). Cytochrome C was downregulated in both the long-lived (p = 0.012; Student’s *t*-test; n = 8) and the short-lived flies (p = 0.006; Student’s *t*-test). GAPDH was used for normalisation. Data are means ±SEM. (C) Production of mitochondrial H_2_O_2_ in the Atg1 over-expressing flies *in vivo* measured by the mass spectrometry probe MitoB. Data are means ±SEM of n = 6 samples (each containing 10 flies). Statistical significance was determined by a one-way ANOVA Tukey-Kramer HSD test (***, p<0.001). (D) The short-lived Atg1 over-expressing flies had increased levels of dihydroxyacetone phosphate, as determined by metabolomics analysis. Statistical significance was calculated by a one-way ANOVA Tukey-Kramer HSD test (***, p<0.001). (E) Oil Red O staining for lipids showed decreased lipid content in autophagy enhanced flies. This lipid loss was very pronounced in the short-lived flies, resulting in almost complete lipid disappearance by day 12.
(PDF)

S6 Fig Metabolomic profiling of the autophagy enhanced flies. (A) Heat map of metabolite profiles for Atg1 over-expressing flies with different longevity phenotypes and controls. Relative metabolite levels shown by colour scale, hierarchical clustering done by Euclidean distance. Hierarchical clustering of raw data revealed that the long-lived fly metabolomics samples clustered closer to controls than short-lived fly samples. (B) Principal component analysis (PCA) of metabolomics data. Unsupervised multivariate analysis indicates consistent metabolic profiles associated with each phenotype. The short-lived flies exhibited a metabolic profile that is very different from controls, as represented by the 1^st^ principal component. The long-lived flies display a metabolic profile that is different from both controls and short-lived flies. Differences between controls and long-lived flies are represented by the 2^nd^ principal component.
(PDF)

S7 Fig Side-by-side comparison of KEGG pathway enrichment for metabolite changes in the mild and strong Atg1 over-expressing flies. Numerous metabolic pathways are affected upon autophagy up-regulation. Metabolic changes are more pronounced in the short-lived flies, compared to the long-lived flies. No unique pathway changes are observed in the long-lived flies. L stands for long-lived (*CSGAL4 tubGAL80*^*ts*^ > *UAS-Atg1(S))*; S for short-lived (*HRGAL4 tubGAL80*^*ts*^ > *UAS-Atg1(S))*; C1 for control 1 (*CSGAL4 tubGAL80*^*ts*^); C2 for control 2 (*UAS-Atg1(S))*.
(PDF)

S8 Fig Volcano plot representing metabolic differences between the long-lived (L) and the short-lived (S) Atg1 over-expressing flies. Metabolites with absolute changes logFC>1 and a FDR significance of <0.05 are marked. Red indicates an increase and blue a decrease in the amount of metabolite.
(PDF)

S1 Data Transcriptomic data for Atg1 over-expressing flies. (XLSX)

S1 Table List of lipid metabolism-related genes that were transcriptionally regulated in the long- and the short-lived Atg1 over-expressing flies. (PDF)
