# Supplementary figures and images for: Fine-tuning autophagy maximises lifespan and is associated with changes in mitochondrial gene expression in Drosophila
Source: PLoS Genet. 2020 Nov 30;16(11):e1009083. doi: 10.1371/journal.pgen.1009083 (PMC7738165; doi:10.1371/journal.pgen.1009083)

**A**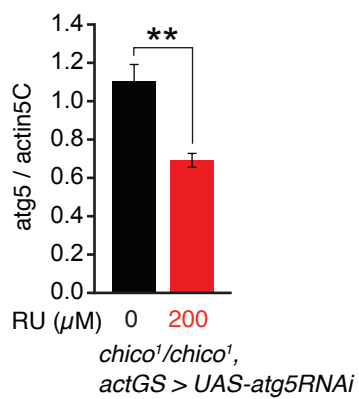**B**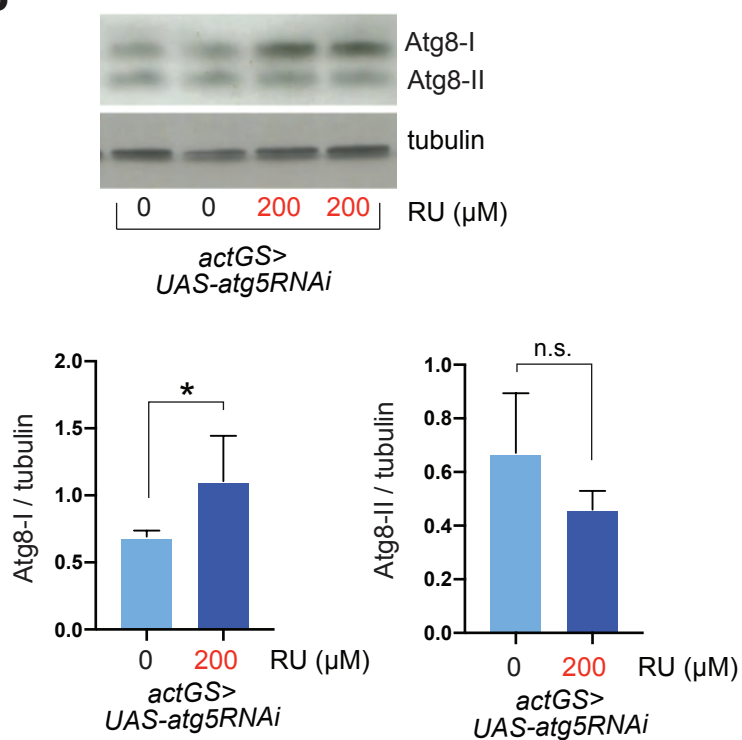**C**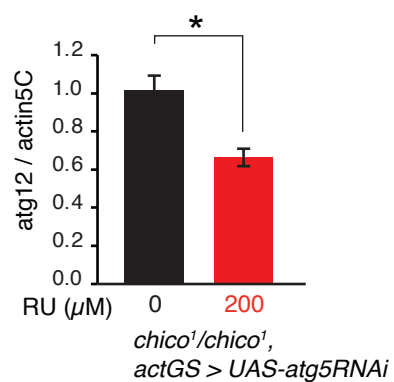**D**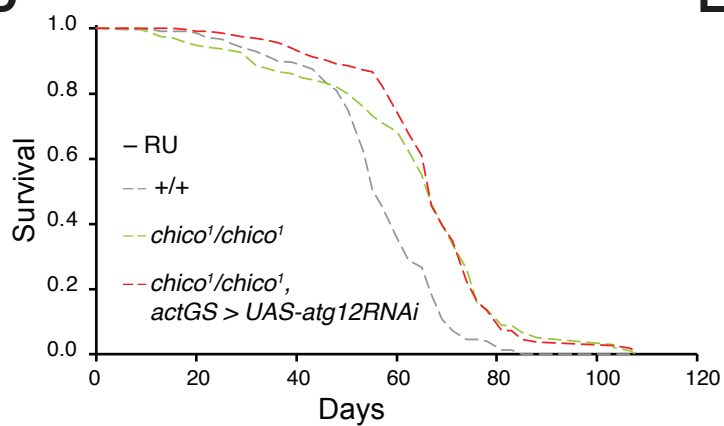**E**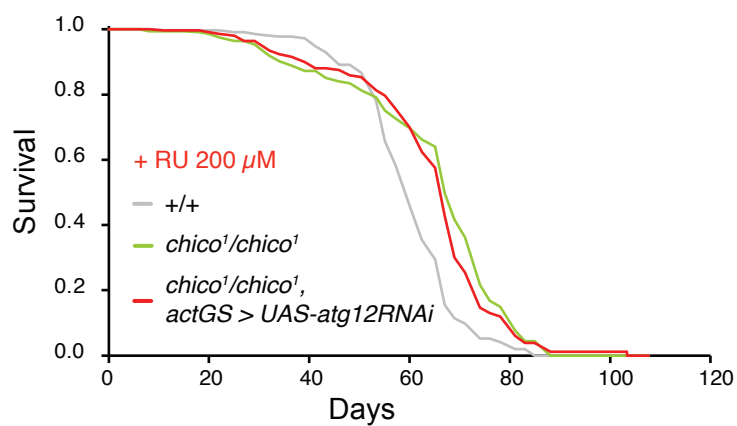**F**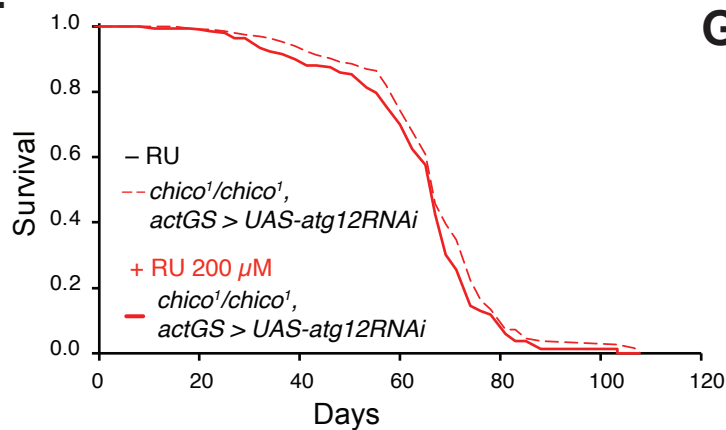**G**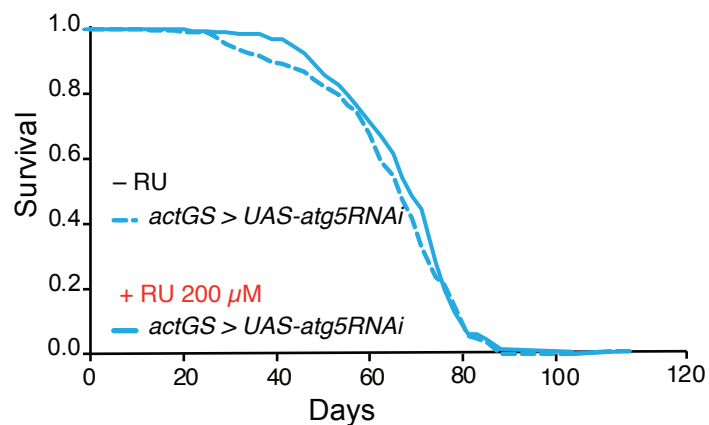

Figure S1.

Supplement: S1 Fig — (A) Confirmation of decreased Atg5 transcription upon overexpression of UAS-Atg5 RNAi. Quantification of Atg5 mRNA levels by qRT-PCR in chico1/chico1 actGS > UAS-atg5RNAi flies ±RU (200 μM). Data are normalised to actin5C, and are means ±SEM of n = 3 samples (p = 0.01, Student’s t-test, ** p<0.01). (B) Western blot analysis showing an increase in Atg8a-I upon inducing down-regulation of Atg5 transcription using RU in actGS > UAS-atg5RNAi flies (p = 0.0002; Student’s t-test). Data are means ±SEM of n = 4 samples. (C) Confirmation of decreased Atg12 transcription upon overexpression of UAS-Atg12 RNAi. Quantification of Atg12 mRNA levels by qRT-PCR in chico1/chico1 actGS > UAS-atg12RNAi flies ±RU (200 μM). Data are normalised to actin5C, and are means ±SEM of n = 3 samples (p = 0.019, Student’s t-test, * p<0.05). (D) Survival of +/+ controls, chico1/chico1, and chico1/chico1 actGS > UAS-atg12RNAi on standard food (–RU). Both chico1/chico1 mutants were longer-lived than the +/+ controls (p<0.0001; log-rank test), but not significantly different from each other (p = 0.65, log-rank test). Survival of +/+ controls and chico1/chico1 are same as in Fig 1. (E) Survival of +/+ controls, chico1/chico1, and chico1/chico1 actGS > UAS-atg12RNAi on RU food. Both chico1/chico1 mutants were longer-lived than the +/+ controls (p<0.0001 and p = 0.001; log-rank test), but not significantly different from each other (p = 0.21, log-rank test). (F) Down-regulation of autophagy by Atg12 RNAi showed a tendency to shorten the lifespan extension of long-lived chico1 null mutants (p = 0.091, log-rank test). Survival curves for chico1/chico1 actGS > UAS-atg12RNAi on control and +RU food (200 μM). n~210 flies per condition for all lifespan experiments. (G) Down-regulation of Atg5 transcription upon RNAi in the adult flies using inducible actGS driver did not affect longevity (p = 0.23, Student’s t-test, n~210 flies per condition). (PDF) [file pgen.1009083.s001.pdf]

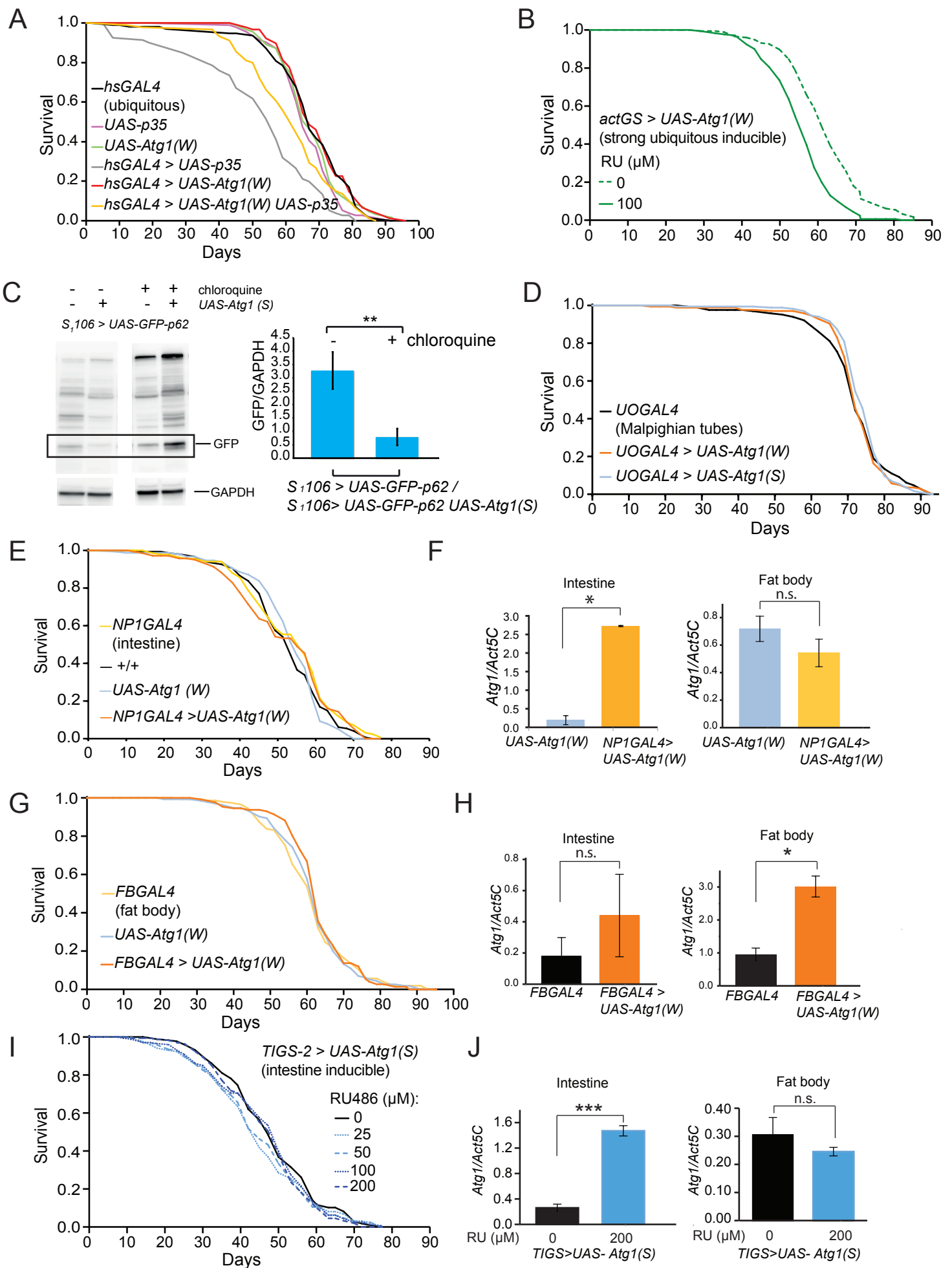

Figure S2.

Supplement: S2 Fig — (A) Constitutive over-expression of UAS-Atg1(W) under weak ubiquitous heat shock hsGAL4 did not alter lifespan compared to driver alone, and lifespan was worsened when overexpression of autophagy was combined with the apoptosis inhibitor p35 (p<0.001, log-rank test comparison of hsGAL4>UAS-Atg1(W)UAS-p35 with hsGAL4). Inhibition of apoptosis shortened lifespan (p<0.001, log-rank test comparison of hsGAL4>UAS-p35 with hsGAL4; n~100 flies per condition). (B) Ubiquitous over-expression of UAS-Atg1(W) under actGS, an inducible ubiquitous GeneSwitch driver, led to lifespan shortening in the presence of 100 μM RU (p<0.001, log-rank test; n~180 flies per condition). (C) GFP-p62 cleavage assay for measurements of autophagy flux demonstrated an apparent decrease in GFP-p62 cleavage in the autophagy flies in the absence of autophagy inhibitor chloroquine but, contrarily, a significant increase in GFP-p62 cleavage under non-saturating levels of chloroquine, suggesting increased flux upon Atg1 overexpression. (p<0.005, Student’s t-test). (D) Over-expression of Atg1 with the Malpighian tubule driver UOGAL4 did not extend lifespan (p = 0.91 and p = 0.38 for the log-rank test comparison of the UOGAL4 control with UOGAL4 > UAS-Atg1(W) and UOGAL4 > UAS-Atg1(S) respectively; n~180 flies per condition). (E) Over-expression of Atg1 using the gut driver NP1GAL4 did not extend lifespan. (p = 0.49, log-rank test comparison of NP1GAL4> UAS-Atg1(W) with the wild-type control, n~160 flies per condition). No significant differences were observed between any of the represented lifespans (p>0.05, log-rank test). NP1GAL4> UAS-Atg1(S) flies were developmentally lethal. (F) qRT-PCR analysis of Atg1 expression in the intestine and fat body of flies from (E) showing a statistically significant increase of Atg1 expression in the intestine of the NP1GAL4 > UAS-Atg1(W) flies (p = 0.047, Student’s t-test; *, p<0.05) but not in the fat body (p = 0.2638, Student’s t-test). (G) Fat body over-expression of [file pgen.1009083.s002.pdf]

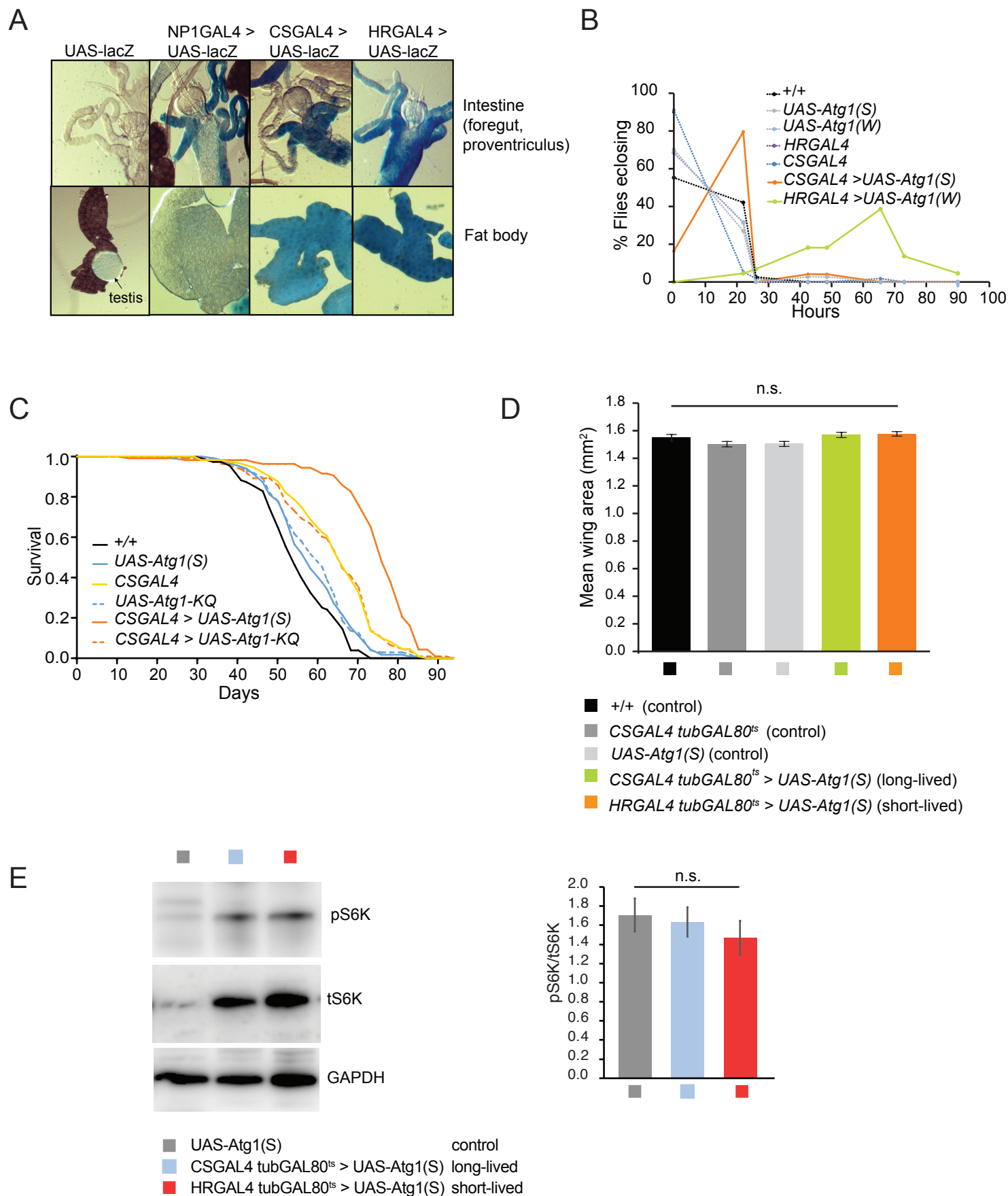

Figure S3.

Supplement: S3 Fig — (A) Representative X-Gal staining for the CSGAL4, HRGAL4 and NP1GAL4 drivers. (B) Development time of the autophagy enhanced flies. Flies with increased autophagy by over-expression of Atg1 driven by CSGAL4 or HRGAL4 displayed delayed egg to adult development. n~100 flies per condition. (C) Over-expression of a kinase dead mutant of Atg1, UAS-Atg1KQ, driven by CSGAL4 did not extend lifespan (p = 0.9936, log-rank test comparison of CSGAL4 > UAS-Atg1KQ with the CSGAL4 driver control). The long-lived autophagy enhanced flies CSGAL4 > UAS-Atg1(S) was significantly longer lived than the CSGAL4 control (p<0.0001, log-rank test). n~120 flies per condition. (D) Body size as inferred from wing area measurement in the Atg1 over-expressing flies. Data are means ±SEM of n = 10 females per genotype. There are no statistically significant differences in wing size among the represented genotypes, as calculated by a one-way ANOVA Tukey-Kramer (HSD). (E) Western blot analysis showing no significant change in pS6K/tS6K in Atg1 over-expressing flies compared to controls (UAS-Atg1(S) compared to CSGAL4 tubGAL80ts > UAS-Atg1(S) p = 0.76, Student’s t-test; and UAS-Atg1(S) compared to HRGAL4 tubGAL80ts > UAS-Atg1(S) p = 0.35, Student’s t-test). (PDF) [file pgen.1009083.s003.pdf]

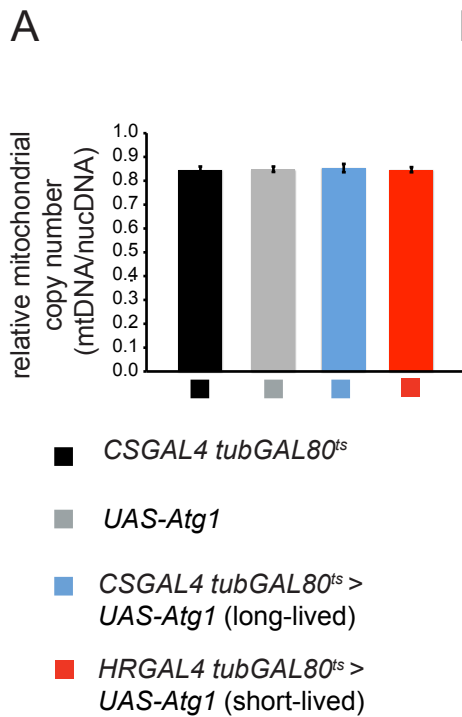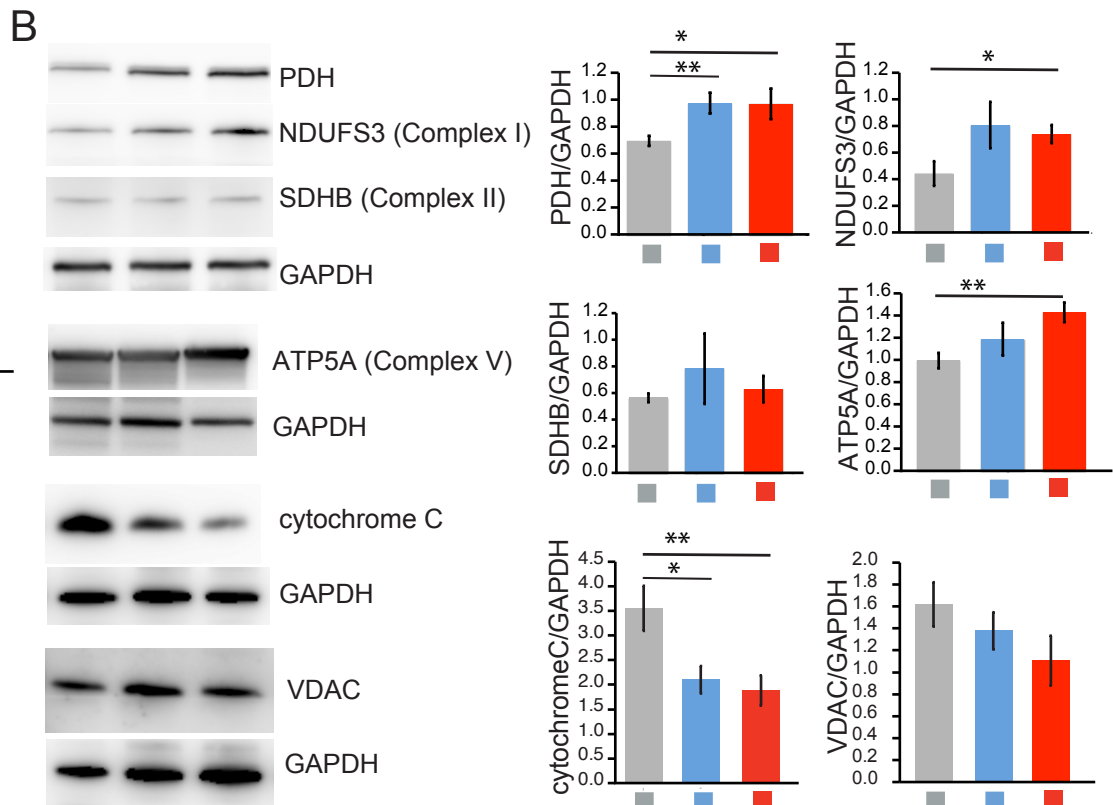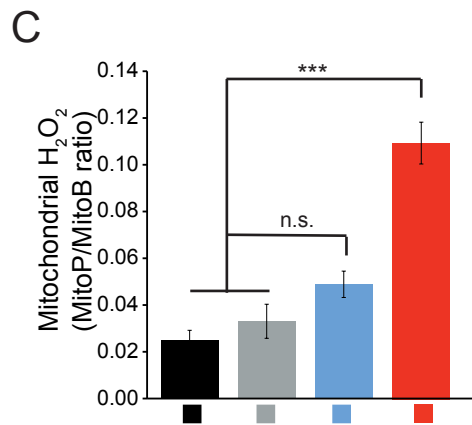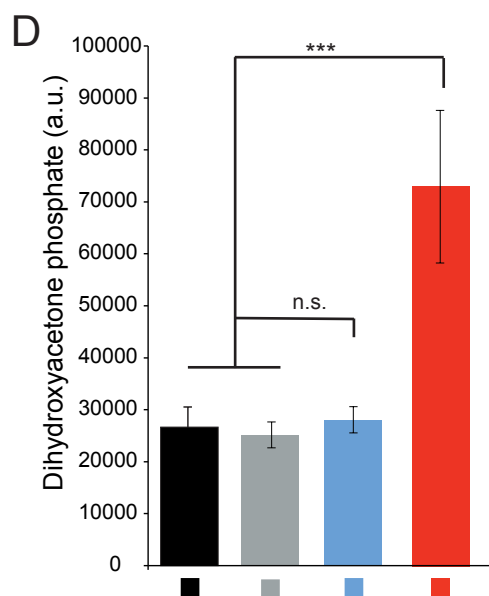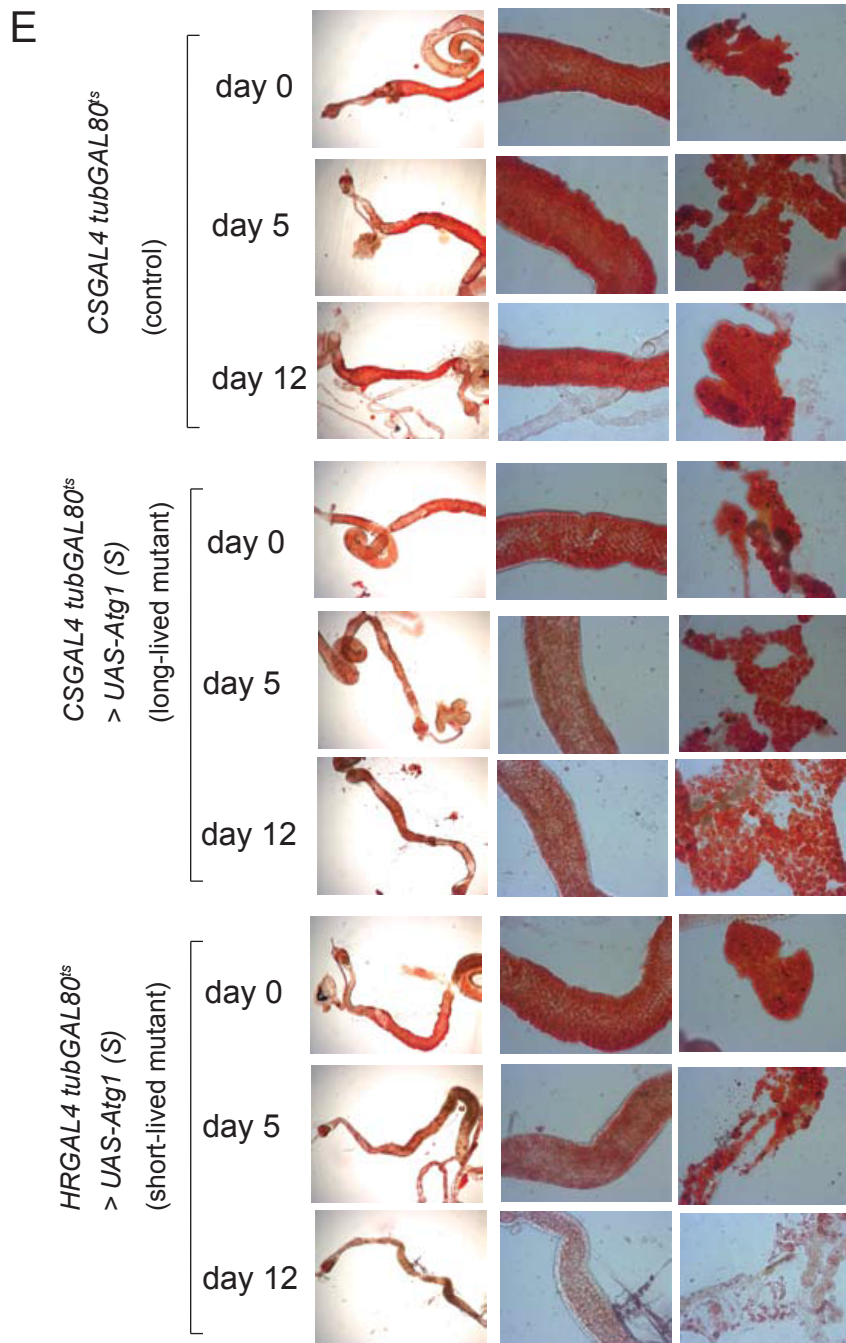

Figure S5.

Supplement: S5 Fig — (A) Relative mitochondrial copy number measured by qRT-PCR showed undetectable differences in Atg1 over-expressing flies. (B) Western blot analysis for different mitochondrial proteins showed increased PDH in the long-lived (p = 0.005, Student’s t-test) and the short-lived flies (p = 0.037, Student’s t-test). Both SDHB levels (p = 0.44 and 0.55 for long-lived and short-lived flies, Student’s t-test) and VDAC levels (p = 0.50 and 0.11 for long-lived and short-lived flies, respectively, Student’s t-test, n = 9) were unaltered. NDUFS3 and ATP5A were increased in short-lived (p = 0.042 and 0.005, Student’s t-test) but not in long-lived flies (p = 0.11 and p = 0.27, Student’s t-test). Cytochrome C was downregulated in both the long-lived (p = 0.012; Student’s t-test; n = 8) and the short-lived flies (p = 0.006; Student’s t-test). GAPDH was used for normalisation. Data are means ±SEM. (C) Production of mitochondrial H2O2 in the Atg1 over-expressing flies in vivo measured by the mass spectrometry probe MitoB. Data are means ±SEM of n = 6 samples (each containing 10 flies). Statistical significance was determined by a one-way ANOVA Tukey-Kramer HSD test (***, p<0.001). (D) The short-lived Atg1 over-expressing flies had increased levels of dihydroxyacetone phosphate, as determined by metabolomics analysis. Statistical significance was calculated by a one-way ANOVA Tukey-Kramer HSD test (***, p<0.001). (E) Oil Red O staining for lipids showed decreased lipid content in autophagy enhanced flies. This lipid loss was very pronounced in the short-lived flies, resulting in almost complete lipid disappearance by day 12. (PDF) [file pgen.1009083.s005.pdf]

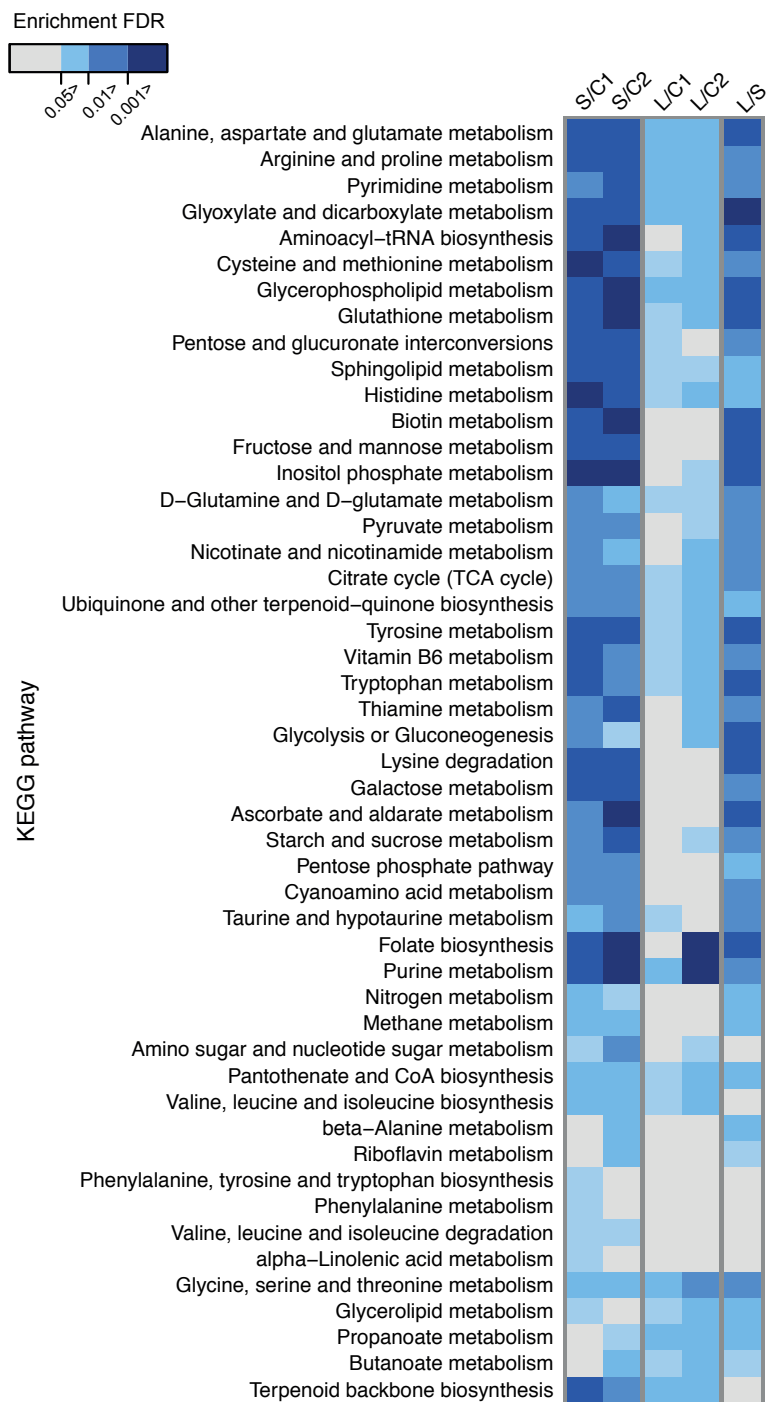

Figure S7

Supplement: S7 Fig — Numerous metabolic pathways are affected upon autophagy up-regulation. Metabolic changes are more pronounced in the short-lived flies, compared to the long-lived flies. No unique pathway changes are observed in the long-lived flies. L stands for long-lived (CSGAL4 tubGAL80ts > UAS-Atg1(S)); S for short-lived (HRGAL4 tubGAL80ts > UAS-Atg1(S)); C1 for control 1 (CSGAL4 tubGAL80ts); C2 for control 2 (UAS-Atg1(S)). (PDF) [file pgen.1009083.s007.pdf]

# Metabolic differences between long-lived (L) and short-lived (S) flies

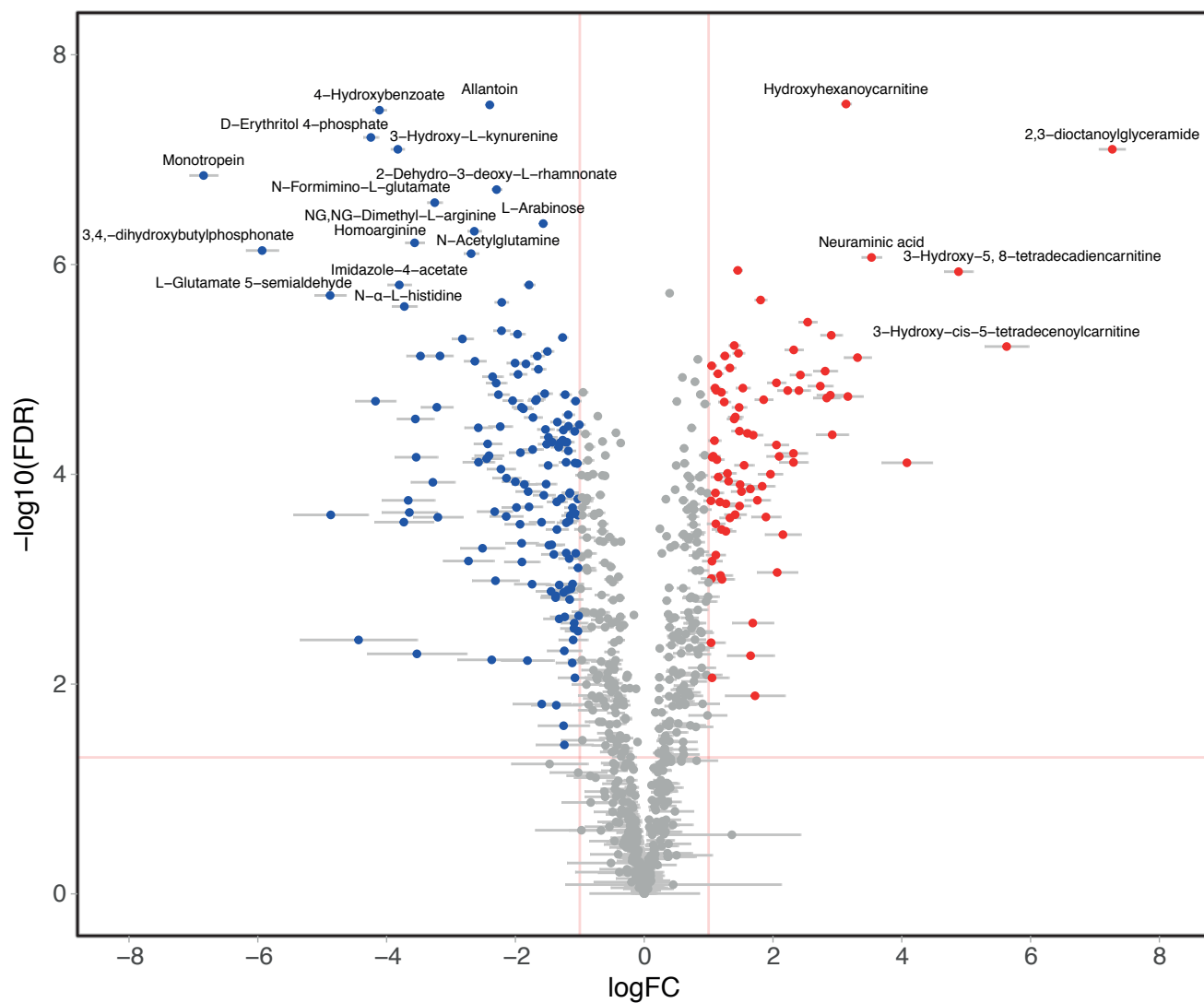

Figure S8.

Supplement: S8 Fig — Metabolites with absolute changes logFC>1 and a FDR significance of <0.05 are marked. Red indicates an increase and blue a decrease in the amount of metabolite. (PDF) [file pgen.1009083.s008.pdf]
