# Supplementary material for: Fine-tuning autophagy maximises lifespan and is associated with changes in mitochondrial gene expression in Drosophila
Source: PLoS Genet. 2020 Nov 30;16(11):e1009083. doi: 10.1371/journal.pgen.1009083 (PMC7738165; doi:10.1371/journal.pgen.1009083)
Supplement: S4 Fig — There was significant over-representation of the differentially expressed genes between the two autophagy enhanced lines. The cut-off point of the adjusted p-value was < 5 x 10^−5^ for the long-lived Atg1 over-expressing flies and <1 x 10^−10^ for the short-lived Atg1-over-expressing flies. (PDF) [file pgen.1009083.s004.pdf]

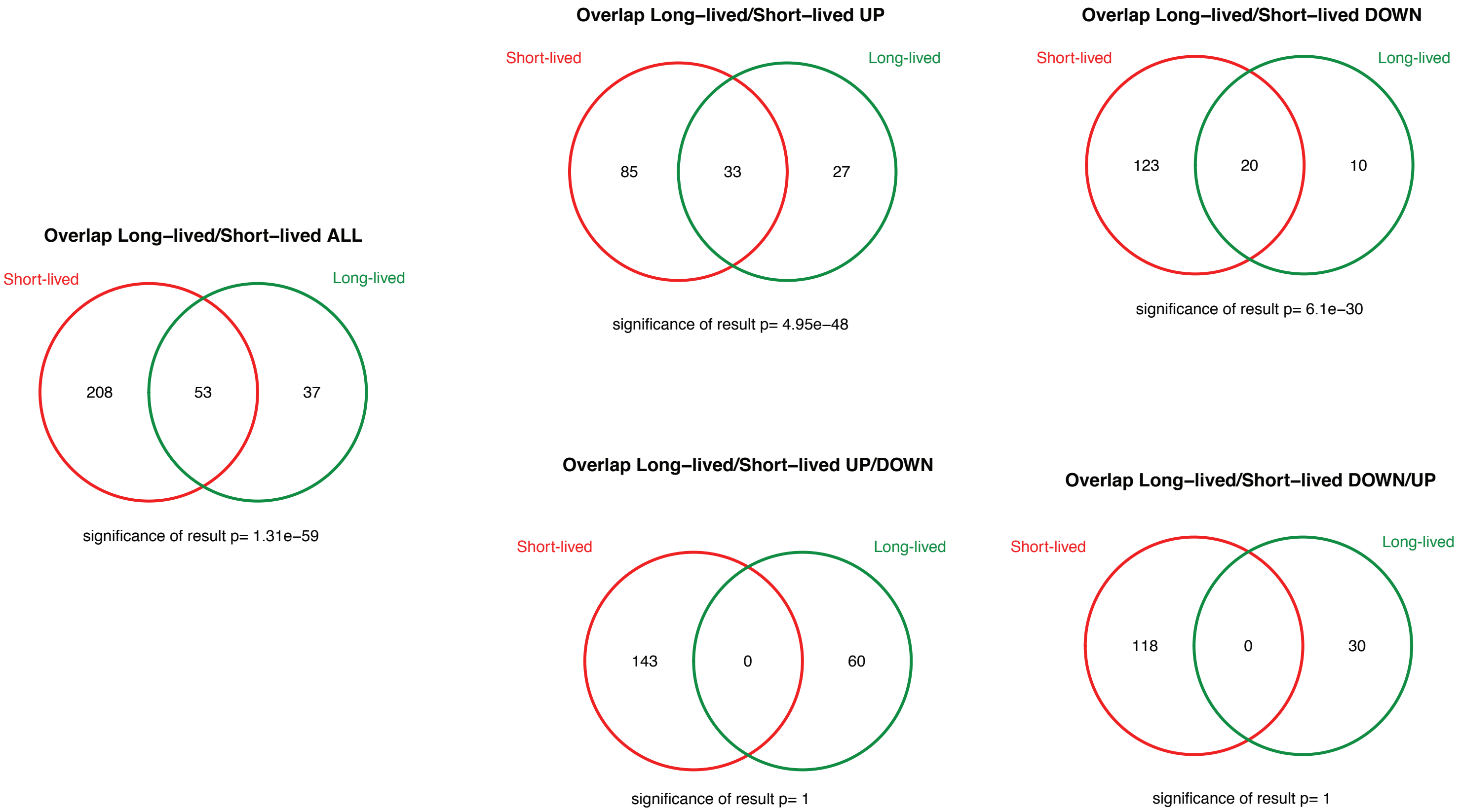

Intersection - up regulated genes

Ensembl.Gene.ID	ID	symbols	Description
FBgn0031945	1627069_at	CG7191	NA
FBgn0259711	1632958_a_at	CG42365	NA
FBgn0039296	1623247_at	CG10420	NA
FBgn0001258	1635227_at	ImpL3	Ecdysone-inducible gene L3
FBgn0033945	1635030_at	CG12868	NA
FBgn0033477	1627152_at	CG12918	NA
FBgn0039788	1634343_at	Rpt6R	NA
FBgn0028523	1635109_at	CG5888	NA
FBgn0030348	1638778_at	CG10352	NA
FBgn0027095	1639410_at	Manf	Mesencephalic astrocyte-derived neurotrophic factor
FBgn0035996	1626220_at	CG3448	NA
FBgn0031547	1624817_at	Sr-CIV	NA
FBgn0020414	1626301_at	Idgf3	Imaginal disc growth factor 3
FBgn0020623	1632701_at	Pal	NA
FBgn0037151	1628660_at	CG7130	NA
FBgn0028695	1637349_at	Rpn1	Rpn1
FBgn0259152	1623888_at	Clbn	Caliban
FBgn0032734	1623841_at	CG15169	NA
FBgn0037391	1633771_s_at	CG2017	NA
FBgn0030614	1623482_at	CG9072	NA
FBgn0028684	1629676_at	Tbp-1	Tat-binding protein-1
FBgn0035165	1626382_s_at	CG13887	NA
FBgn0036126	1628683_at	CG6272	NA
FBgn0020369	1639784_at	Pros45	Pros45
FBgn0036210	1629287_at	CG14130	NA
FBgn0031267	1628514_at	Ipk2	Ipk2
FBgn0038179	1635294_at	CG9312	NA
FBgn0029937	1635581_at	CG8300	NA
FBgn0050022	1628558_at	CG30022	NA
FBgn0033672	1627697_at	rho-7	rhomboid-7
FBgn0036196	1640228_at	CG11658	NA
FBgn0261396	1628269_at	Rpn3	Regulatory particle non-ATPase 3
FBgn0004595	1635500_a_at	pros	prospero

Intersection - down regulated genes

Ensembl.Gene.ID	ID	symbols	Description
FBgn0010425	1637492_at	epsilonTry	epsilonTrypsin
FBgn0039471	1635868_at	CG6295	NA
FBgn0023197	1624824_at	Jon74E	Jonah 74E
FBgn0002939	1632705_at	ninaD	neither inactivation nor afterpotential D
FBgn0033774	1638361_at	CG12374	NA
FBgn0039342	1634240_at	CG5107	NA
FBgn0039472	1629308_at	CG17192	NA
FBgn0039311	1623265_at	CG10513	NA
FBgn0030098	1634076_at	CG12057	NA
FBgn0032913	1635498_at	CG9259	NA
FBgn0039325	1627144_at	CG10560	NA
FBgn0038718	1627080_at	CG17752	NA
FBgn0038484	1640355_at	CG5246	NA
FBgn0039470	1632215_at	CG6296	NA
FBgn0039114	1636510_a_at	Lsd-1	Lipid storage droplet-1
FBgn0032068	1625374_at	CG9466	NA
FBgn0036738	1629476_at	CG7542	NA
FBgn0010357	1630320_at	betaTry	betaTrypsin
FBgn0032049	1635453_at	Bace	beta-site APP-cleaving enzyme
FBgn0030594	1631420_at	CG9509	NA

Figure S4.
