# Supplementary material for: Fine-tuning autophagy maximises lifespan and is associated with changes in mitochondrial gene expression in Drosophila
Source: PLoS Genet. 2020 Nov 30;16(11):e1009083. doi: 10.1371/journal.pgen.1009083 (PMC7738165; doi:10.1371/journal.pgen.1009083)
Supplement: S6 Fig — (A) Heat map of metabolite profiles for Atg1 over-expressing flies with different longevity phenotypes and controls. Relative metabolite levels shown by colour scale, hierarchical clustering done by Euclidean distance. Hierarchical clustering of raw data revealed that the long-lived fly metabolomics samples clustered closer to controls than short-lived fly samples. (B) Principal component analysis (PCA) of metabolomics data. Unsupervised multivariate analysis indicates consistent metabolic profiles associated with each phenotype. The short-lived flies exhibited a metabolic profile that is very different from controls, as represented by the 1st principal component. The long-lived flies display a metabolic profile that is different from both controls and short-lived flies. Differences between controls and long-lived flies are represented by the 2nd principal component. (PDF) [file pgen.1009083.s006.pdf]

A

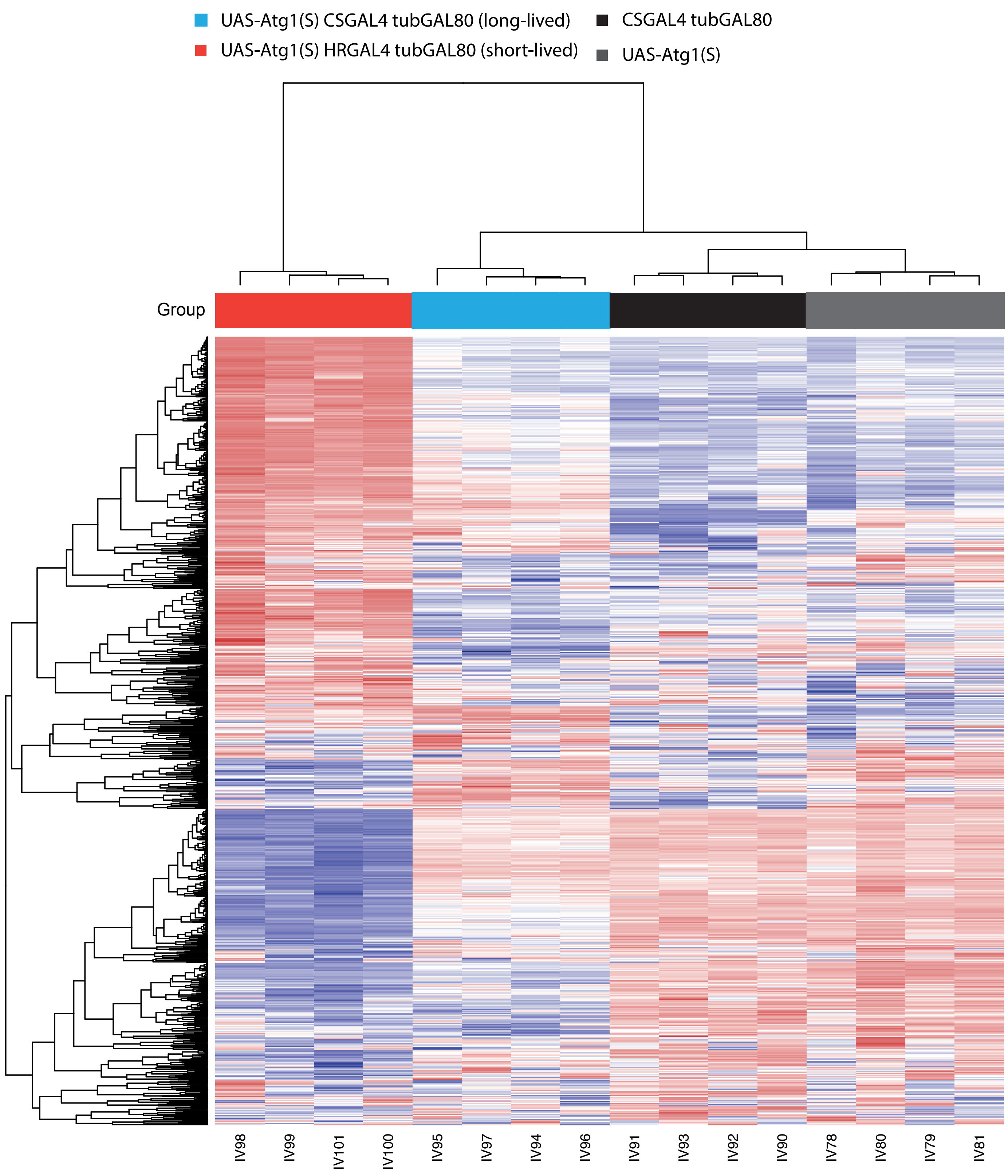
UAS-Atg1(S) CSGAL4 tubGAL80 (long-lived)
UAS-Atg1(S) HRGAL4 tubGAL80 (short-lived)
CSGAL4 tubGAL80
UAS-Atg1(S)
Group
IV98
IV99
IV101
IV100
IV95
IV97
IV94
IV96
IV91
IV93
IV92
IV90
IV78
IV80
IV79
IV81

B

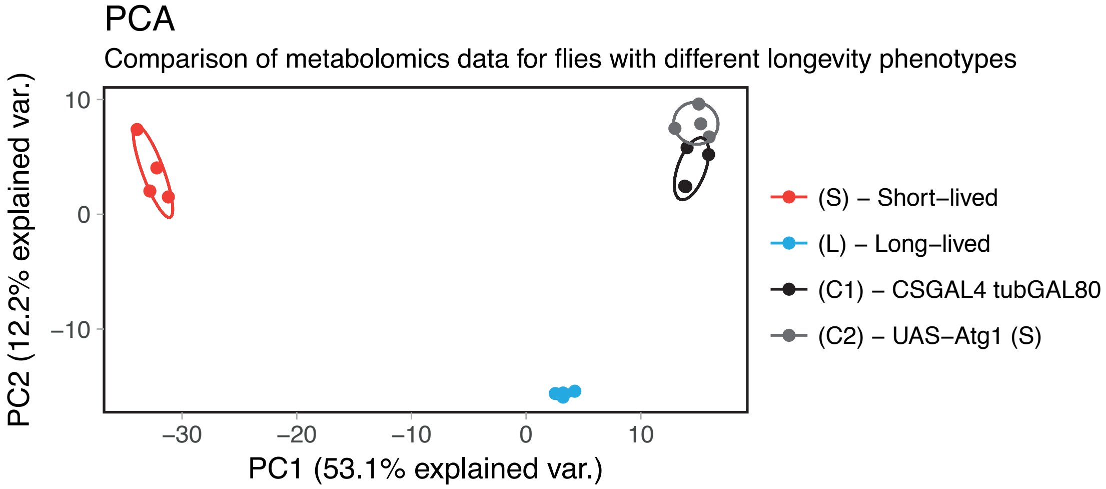
PCA
Comparison of metabolomics data for flies with different longevity phenotypes
PC2 (12.2% explained var.)
10
0
−10
−30
−20
−10
0
10
PC1 (53.1% explained var.)
(S) – Short–lived
(L) – Long–lived
(C1) – CSGAL4 tubGAL80
(C2) – UAS–Atg1 (S)

Figure S6
