# Supplementary material for: Fine-tuning autophagy maximises lifespan and is associated with changes in mitochondrial gene expression in Drosophila
Source: PLoS Genet. 2020 Nov 30;16(11):e1009083. doi: 10.1371/journal.pgen.1009083 (PMC7738165; doi:10.1371/journal.pgen.1009083)
Supplement: S1 Table — (PDF) [file pgen.1009083.s010.pdf]

long-lived (UAS-Atg1(W) CSGAL4 tubGAL80) vs GAL4 driver - down-regulated genes

Ensembl.Gene.II	ID	logFC	AveExpr	P.Value	adj.P.Val	driverControl	longLivedMuta	symbols	Description		
FBgn0039471	1635868_at	-4.788	11.466	1.60E-12	5.30E-09	13.44	8.53	CG6295	CG6295	NA	lipase
FBgn0039472	1629308_at	-6.562	9.631	5.50E-10	6.00E-07	12.47	5.59	CG17192	CG17192	NA	lipase
FBgn0039470	1632215_at	-3.602	9.197	2.60E-08	7.10E-06	10.95	6.92	CG6296	CG6296	NA	lipase
FBgn0039114	1636510_a_at	-1.162	11.668	2.60E-08	7.20E-06	12.31	10.86	Lsd-1	CG10374	Lipid storage droplet-1	lipid droplet
FBgn0031069	1629413_at	-0.777	11.919	2.50E-07	2.80E-05	12.15	11.52	CG12703	CG12703	NA	peroxisome
FBgn0031533	1633709_at	-1.389	8.982	8.80E-06	0.00027	9.62	8.22	CG2772	CG2772	NA	lipase
FBgn0031449	1639286_s_at	-0.696	10.259	9.90E-06	3.00E-04	10.54	9.87	CG31689	CG31689	NA	lipid metabolism
FBgn0038795	1638038_at	-0.745	8.822	1.00E-05	3.00E-04	9.13	8.38	CG4335	CG4335	NA	b-oxidation*
FBgn0030013	1628080_at	-0.832	9.757	1.00E-05	3.00E-04	10.18	9.22	GIIIspla2	CG1583	NA	lipase
FBgn0036996	1636583_at	-1.648	12.013	1.40E-05	0.00037	12.68	11.14	CG5932	CG5932	NA	lipase
FBgn0030608	1637359_at	-0.585	11.382	1.80E-05	0.00043	11.64	11.05	Lsd-2	CG9057	Lipid storage droplet-2	lipase
FBgn0036449	1640754_at	-0.517	10.366	1.90E-05	0.00046	10.52	10.11	bmm	CG5295	brummer	lipase
FBgn0027842	1626147_s_at	-0.897	11.774	3.00E-05	0.00062	12.26	11.22	CPTI	CG12891	NA	b-oxidation*
FBgn0035169	1629606_at	-0.408	9.325	3.60E-05	7.00E-04	9.47	9.09	CG13890	CG13890	NA	b-oxidation
FBgn0051414	1630993_at	-0.537	6.305	3.70E-05	0.00071	6.33	6.15	CG31414	CG31414	NA	sphingolipid metabolism

long-lived (UAS-Atg1(W) CSGAL4 tubGAL80) vs GAL4 driver - up-regulated genes

Ensembl.Gene.II	ID	logFC	AveExpr	P.Value	adj.P.Val	driverControl	longLivedMuta	symbols	Description		
FBgn0051148	1637647_at	0.792	11.691	2.90E-07	3.00E-05	11.37	12.18	CG31148	CG31148	NA	sphingolipid metabolism
FBgn0261283	1628292_s_at	0.38	10.355	5.30E-06	2.00E-04	10.27	10.56	HLH106	CG8522	Helix loop helix protein 106	SREBP pathway
FBgn0031381	1625334_at	0.668	11.241	1.10E-05	0.00032	11	11.59	Npc2a	CG7291	Niemann-Pick type C-2a	sterol traffic
FBgn0025373	1628428_at	0.508	10.3	2.30E-05	0.00051	10.12	10.58	Fpps	CG12389	Farnesyl pyrophosphate synthase	isoprenoid biosynthesis

short-lived (UAS-Atg1(S) HRGAL4 tubGAL80) vs GAL4 driver - down-regulated genes

Ensembl.Gene.II	ID	logFC	AveExpr	P.Value	adj.P.Val	driverControl	longLivedMuta	symbols	Description		
FBgn0004047	1631419_at	-5.551	12.341	8.60E-20	4.30E-16	14.15	8.6	Yp3	CG11129	Yolk protein 3	lipase
FBgn0039114	1636510_a_at	-3.968	10.931	1.30E-16	1.80E-13	12.23	8.26	Lsd-1	CG10374	Lipid storage droplet-1	lipid droplet
FBgn0039470	1632215_at	-6.677	7.998	6.60E-15	1.50E-12	10.56	3.89	CG6296	CG6296	NA	lipase
FBgn0039472	1629308_at	-6.841	10.076	5.90E-14	7.00E-12	12.17	5.33	CG17192	CG17192	NA	lipase
FBgn0027571	1624549_at	-1.383	13.269	9.00E-14	8.70E-12	13.69	12.31	CG3523	CG3523	NA	fatty acid synthesis
FBgn0036996	1636583_at	-5.701	10.656	1.00E-13	9.40E-12	12.42	6.72	CG5932	CG5932	NA	lipase
FBgn0005391	1623655_at	-1.98	13.537	1.30E-13	1.10E-11	14.16	12.18	Yp2	CG2979	Yolk protein 2	lipase
FBgn0030608	1637359_at	-1.527	11.07	6.80E-13	3.40E-11	11.56	10.03	Lsd-2	CG9057	Lipid storage droplet-2	lipid droplet
FBgn0031362	1634296_s_at	-1.674	10.615	6.90E-13	3.40E-11	11.13	9.45	CG17646	CG17646	NA	lipid metabolism
FBgn0031533	1633709_at	-4.116	8.142	7.80E-13	3.70E-11	9.13	5.01	CG2772	CG2772	NA	lipase
FBgn0038795	1638038_at	-1.904	8.575	8.30E-13	3.80E-11	9.14	7.24	CG4335	CG4335	NA	b-oxidation*
FBgn0037782	1633050_at	-5.248	9.814	1.40E-12	5.50E-11	11.26	6.02	Npc2d	CG12813	Niemann-Pick type C-2d	sterol traffic
FBgn0039471	1635868_at	-6.813	11.155	2.90E-12	9.40E-11	13.34	6.53	CG6295	CG6295	NA	lipase
FBgn0039755	1638528_at	-0.865	10.881	5.40E-12	1.40E-10	11.2	10.33	CG15531	CG15531	NA	lipid metabolism
FBgn0004045	1629545_at	-0.789	14.207	6.00E-12	1.50E-10	14.45	13.66	Yp1	CG2985	Yolk protein 1	lipase
FBgn0021765	1634433_at	-0.81	11.986	1.40E-11	2.80E-10	12.22	11.41	scu	CG7113	scully	b-oxidation
FBgn0035811	1626566_at	-1.199	11.786	2.30E-11	4.10E-10	12.22	11.02	CG12262	CG12262	NA	b-oxidation
FBgn0034629	1633476_at	-1.58	10.269	3.30E-11	5.60E-10	10.66	9.08	Acox57D-d	CG9709	acyl-Coenzyme A oxidase at 57D distal	b-oxidation
FBgn0031813	1637461_at	-1.07	11.081	4.20E-11	6.60E-10	11.38	10.31	CG9527	CG9527	NA	b-oxidation
FBgn0004797	1636579_s_at	-1.117	9.036	4.40E-11	6.90E-10	9.41	8.29	mdy	CG31991	midway	lipid synthesis
FBgn0032029	1628290_s_at	-0.978	10.306	4.50E-11	7.00E-10	10.49	9.51	CG17292	CG17292	NA	lipase

short-lived (UAS-Atg1(S) HRGAL4 tubGAL80) vs GAL4 driver - up-regulated genes

Ensembl.Gene.II	ID	logFC	AveExpr	P.Value	adj.P.Val	driverControl	longLivedMuta	symbols	Description		
FBgn0261283	1628292_s_at	1.278	10.678	1.70E-15	7.00E-13	10.23	11.51	HLH106	CG8522	Helix loop helix protein 106	SREBP pathway
FBgn0039474	1636343_at	3.334	9.391	3.30E-14	4.80E-12	8.47	11.8	CG6283	CG6283	NA	lipase
FBgn0031881	1629040_at	1.351	8.725	9.10E-14	8.70E-12	8.13	9.48	CG3476	CG3476	NA	b-oxidation
FBgn0033226	1638693_s_at	1.024	11.454	4.70E-12	1.30E-10	11.06	12.09	CG1882	CG1882	NA	lipase
FBgn0041342	1629930_s_at	0.974	9.723	5.00E-11	7.60E-10	9.42	10.4	Cct1	CG1049	CTP:phosphocholine cytidylyl transfer.	SREBP pathway

Table S1.
